# NASA’s first ground-based Galactic Cosmic Ray Simulator: Enabling a new era in space radiobiology research

**DOI:** 10.1371/journal.pbio.3000669

**Published:** 2020-05-19

**Authors:** Lisa C. Simonsen, Tony C. Slaba, Peter Guida, Adam Rusek

**Affiliations:** 1 NASA Langley Research Center, Hampton, Virginia, United States of America; 2 Brookhaven National Laboratory, Brookhaven, New York, United States of America; National Cancer Institute, UNITED STATES

## Abstract

With exciting new NASA plans for a sustainable return to the moon, astronauts will once again leave Earth’s protective magnetosphere only to endure higher levels of radiation from galactic cosmic radiation (GCR) and the possibility of a large solar particle event (SPE). Gateway, lunar landers, and surface habitats will be designed to protect crew against SPEs with vehicle optimization, storm shelter concepts, and/or active dosimetry; however, the ever penetrating GCR will continue to pose the most significant health risks especially as lunar missions increase in duration and as NASA sets its aspirations on Mars. The primary risks of concern include carcinogenesis, central nervous system (CNS) effects resulting in potential in-mission cognitive or behavioral impairment and/or late neurological disorders, degenerative tissue effects including circulatory and heart disease, as well as potential immune system decrements impacting multiple aspects of crew health. Characterization and mitigation of these risks requires a significant reduction in the large biological uncertainties of chronic (low-dose rate) heavy-ion exposures and the validation of countermeasures in a relevant space environment. Historically, most research on understanding space radiation-induced health risks has been performed using acute exposures of monoenergetic single-ion beams. However, the space radiation environment consists of a wide variety of ion species over a broad energy range. Using the fast beam switching and controls systems technology recently developed at the NASA Space Radiation Laboratory (NSRL) at Brookhaven National Laboratory, a new era in radiobiological research is possible. NASA has developed the “GCR Simulator” to generate a spectrum of ion beams that approximates the primary and secondary GCR field experienced at human organ locations within a deep-space vehicle. The majority of the dose is delivered from protons (approximately 65%–75%) and helium ions (approximately 10%–20%) with heavier ions (Z ≥ 3) contributing the remainder. The GCR simulator exposes state-of-the art cellular and animal model systems to 33 sequential beams including 4 proton energies plus degrader, 4 helium energies plus degrader, and the 5 heavy ions of C, O, Si, Ti, and Fe. A polyethylene degrader system is used with the 100 MeV/n H and He beams to provide a nearly continuous distribution of low-energy particles. A 500 mGy exposure, delivering doses from each of the 33 beams, requires approximately 75 minutes. To more closely simulate the low-dose rates found in space, sequential field exposures can be divided into daily fractions over 2 to 6 weeks, with individual beam fractions as low as 0.1 to 0.2 mGy. In the large beam configuration (60 × 60 cm^2^), 54 special housing cages can accommodate 2 to 3 mice each for an approximately 75 min duration or 15 individually housed rats. On June 15, 2018, the NSRL made a significant achievement by completing the first operational run using the new GCR simulator. This paper discusses NASA’s innovative technology solution for a ground-based GCR simulator at the NSRL to accelerate our understanding and mitigation of health risks faced by astronauts. Ultimately, the GCR simulator will require validation across multiple radiogenic risks, endpoints, doses, and dose rates.

## Abstract 2

This study describes how NASA’s new earth-based galactic cosmic ray simulator is being used to accelerate our understanding of the effects of space radiation exposure on astronauts and to validate countermeasures for exploration missions. For the first time, research teams can study mixed field ion and dose rate effects in a simulated space environment.

## Introduction

The goal of NASA’s space radiation research program is to enable the human exploration of space beyond low-Earth-orbit (LEO) with acceptable risk and mitigation strategies in place to ensure the health, safety, and productivity of our crew. With quick-paced Artemis Moon-to-Mars program goals, NASA is committed to landing American astronauts on the Moon by 2024 and establishing sustainable lunar missions by 2028 in preparation for our next giant leap, sending astronauts to Mars (https://www.nasa.gov/what-is-artemis). The mitigation of health risks from both intermittent solar particle events (SPEs) and chronic galactic cosmic radiation (GCR) will become more challenging as crew leave the protection provided by the Earth’s magnetosphere. Although improved storm shelter designs, space weather forecasting, and operational dosimetry can significantly reduce the risk of acute radiation syndromes from a large SPE, the potential for in-mission health and performance decrements as well as the risk of long-term health consequences from chronic exposure to GCR remain a significant challenge to characterize and mitigate for long-duration missions [1,2]. Physical shielding strategies designed to significantly reduce the health risks from GCR become prohibitively costly because of the high charge (Z) and energy (E) of the primary constituent particles, referred to as high charge and high energy (HZE) particles, and from the production of secondary protons, neutrons, and heavier fragments produced as a result of interactions with spacecraft shielding and human tissues [3]. These heavy ions, low-energy protons, and helium ions are highly ionizing forms of radiation with spatial distributions of radiation deposition, or energy loss, in tissue that are very different from those caused by common forms of radiation found on Earth, namely, x-rays and gamma rays. The pattern of energy loss from highly ionizing particles is characterized by a dense track of ionizations and atomic excitations, along a straight line corresponding to the particle’s trajectory, and a penumbra of higher-energy electrons that may extend hundreds of microns from the particle’s path in tissue. The core track, which can be many centimeters in length, produces extremely large clusters of ionizations within a few nanometers, which is qualitatively distinct from the electron energy depositions more uniformly distributed by x-rays or gamma rays. These differences in the temporal and spatial deposition of energy in tissues from space radiation impart unique biological damage to biomolecules and cells compared with terrestrial radiation, which, for a given dose, is much more damaging. The biological effects of these ions are poorly understood.

Characterizing the biological response of cells, tissues, and animal models in a relevant space environment and understanding the qualitative differences in biological damage compared with terrestrial radiation is essential in establishing and/or validating models of risk, establishing permissible exposure limits (PELs), and developing effective countermeasure strategies to enable safe, long-duration space travel. In 2003, NASA commissioned the NASA Space Radiation Laboratory (NSRL) at Brookhaven National Laboratory (BNL) with a dedicated beamline and laboratory space to conduct ground-based, heavy-ion research. The facility is capable of supplying particles from protons to gold. Available beam energies range from 50 to 2,500 MeV for protons and 50 to 1,500 MeV/n for ions between helium and iron. Heavier ions from charge Z = 27 to 79 are limited to approximately 350 to 500 MeV/n (https://www.bnl.gov/nsrl/). Over the last decade, facility investments in ion source and controls technology enabled the fast switching of ion-energy beam combinations from an automated list of hardware settings in a predetermined sequential order to more closely approximate exposures in space. With input from an international community of physicists and radiobiologists [4], NASA has developed a “GCR Simulator” at BNL for principal investigator (PI)–led research studies. In 2018, the NSRL GCR Simulator was commissioned to simulate the shielded radiation environment encountered by an astronaut within a typical exploration vehicle. This new capability is currently being used to inform our understanding of space radiation effects on the risk of cancer, CNS decrements, and cardiovascular disease endpoints and to establish a ground-based, space-relevant environment to test countermeasure efficacy. Research is progressing to quantify mixed-field radiation quality effects, dose, and dose-rate effects in multiple cellular and animal model systems. Modelling approaches are being developed to leverage historical data sets of acute single-ion experiments to determine simple additivity, synergistic, or antagonistic effects on risk characterization in a mixed field as well as to quantify the impact of acute versus chronic exposures on existing data sets.

## Methods and models

**Fig 1:**
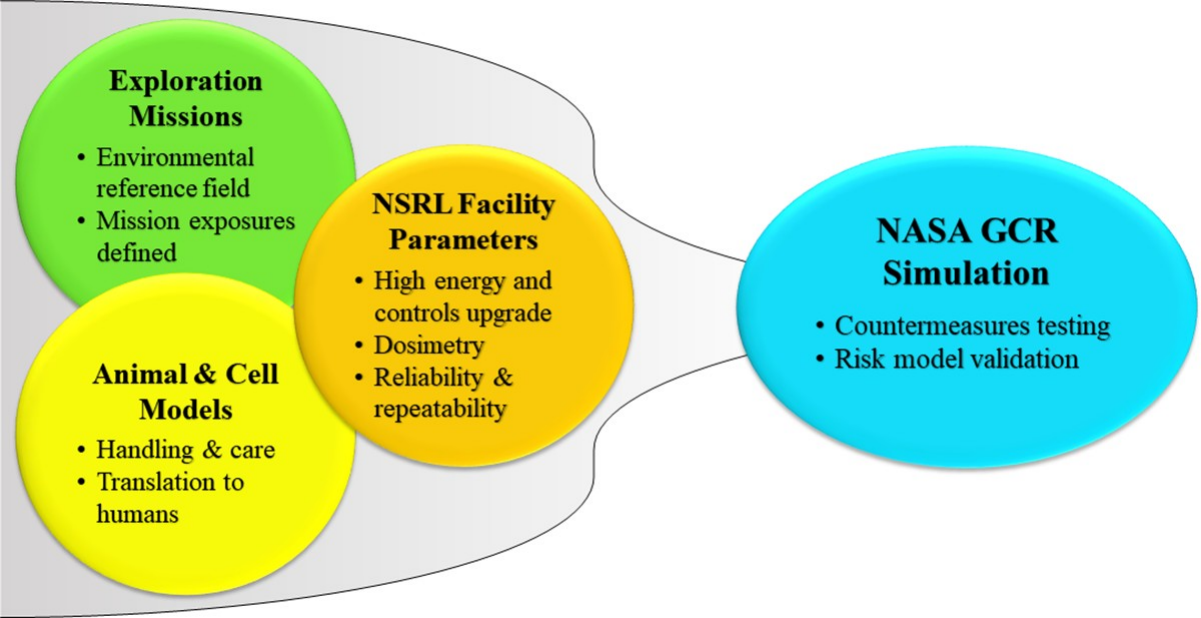
Three key areas that must be developed together to ultimately provide the GCR simulator at NSRL. Development focused on establishing irradiation requirements and balancing facility capabilities and limitations, including constraints imposed by animal and cellular model systems. GCR, galactic cosmic radiation; NSRL, NASA Space Radiation Laboratory.

Three key areas were considered in concert to design the GCR simulator including (1) defining mission relevant radiation environments and resulting exposures, (2) accounting for NSRL facility limitations and completing required hardware and software upgrades, and (3) accounting for constraints imposed by the care and handling of animals and cellular studies as shown in Fig 1. The design of the irradiation field must consider aspects important to each of the major health risk areas. For example, a simpler particle field may be adequate to characterize cancer compared with a more complex field that may or may not be required to characterize CNS decrements. The validation of countermeasures in what is defined as a “space relevant environment” may require additional complexities in field description.

This paper describes each of the major aspects in the development of the capability and briefly discusses the first radiobiology experiments performed in 2018. Although some of the strategies and implementation are specific to NSRL, they can be applied in the broader context to support the development of a GCR simulator capability at other research facilities.

### Development approach

#### Galactic cosmic ray environment

Galactic cosmic rays originate outside the solar system and are likely formed by explosive events such as supernova. They consist of the nuclei of the chemical elements, from hydrogen to uranium, which have been accelerated to extremely high energies outside our solar system. GCR ions are highly penetrating and form a continuous background of radiation in space. Because of a sharp decline in abundance for ions heavier than iron (Z = 26), elements up to nickel (Z = 28) are of most concern. The energy spectra of all GCR particles are very broad with the region extending from approximately 10 MeV/n to 50 GeV/n being of primary importance to space applications [3,5,6]. Within our solar system, the solar wind modulates the flux of galactic cosmic rays over an approximate 11-year cycle with an intensity that is inversely correlated with solar activity. During phases of higher solar activity, the GCR intensity is at a minimum, whereas at solar minimum, the GCR intensity is maximal. At solar maximum, the cosmic ray flux is decreased by a factor of 3 to 4 compared to solar minimum [3], whereas exposure estimates behind typical spacecraft shielding are reduced by roughly a factor of 2 [7,8].

**Fig 2:**
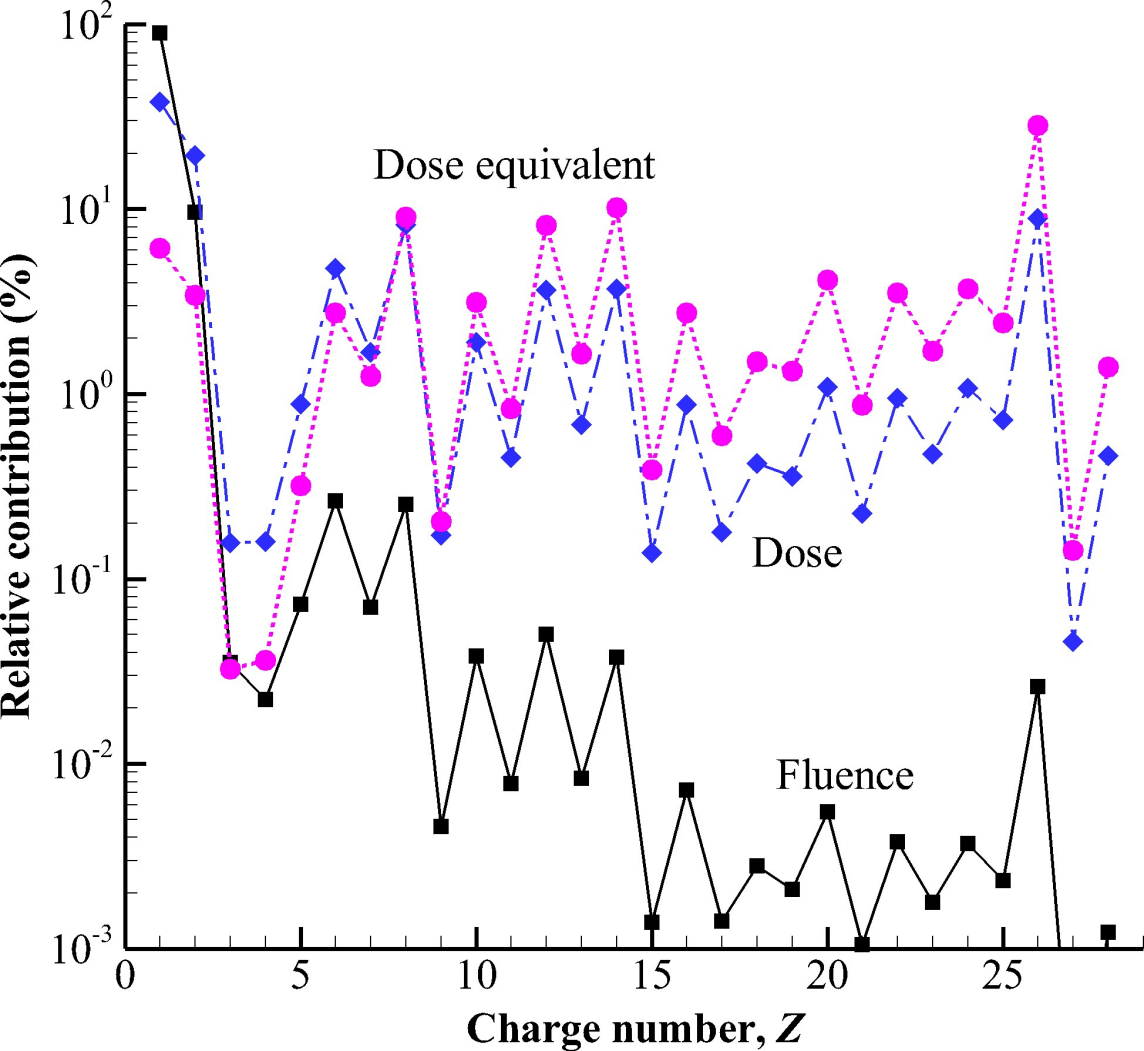
Relative contribution to fluence (squares), dose (diamonds), and dose equivalent (circles) of different elements in the free-space GCR environment during solar minimum conditions (June 1976) as described by the Badhwar–O'Neill 2010 GCR model [14] (Adapted from Durante and Cucinotta [3]). Plot data available in S1 Data. GCR, galactic cosmic radiation.

In free space, the relative contribution of the different elements comprising GCR in flux, dose, and dose equivalent is shown in Fig 2. The most abundant GCR particle types include hydrogen (Z = 1), helium (Z = 2), carbon (Z = 6), oxygen (Z = 8), neon (Z = 10), silicon (Z = 14), calcium (Z = 20), and iron (Z = 26). Protons account for nearly 87% of the total flux, helium ions account for approximately 12%, and the remaining heavy ions account for less than 1% of the total flux [9]. Multiplying the abundance by charge squared (Z^2^) provides an estimate of the relative contribution to dose [10]. Further weighting by quality factor, QF, which relates the absorbed dose to the biological effectiveness of the particle producing the dose [11], provides an estimate of dose equivalent (mSv) used to represent radiation-induced cancer and genetic damage (stochastic health effects). Even though the number of HZE particles is relatively small, they contribute approximately 89% of the total dose equivalent (mSv) in free space [12]. Of the HZE particles, iron is the largest contributor to GCR dose equivalent, making up approximately 26% of the total [13]. Although dose equivalent provides an important quantity of biological damage for carcinogenesis, the relative biological effectiveness of GCR particles inducing degenerative tissue effects including cardiovascular diseases, neurodegenerative conditions, and the potential for early decrements to the CNS are largely unknown. Iron (Fe) is one of the most widely used heavy ions for radiobiological studies, creating a large historical database of results.

**Fig 3:**
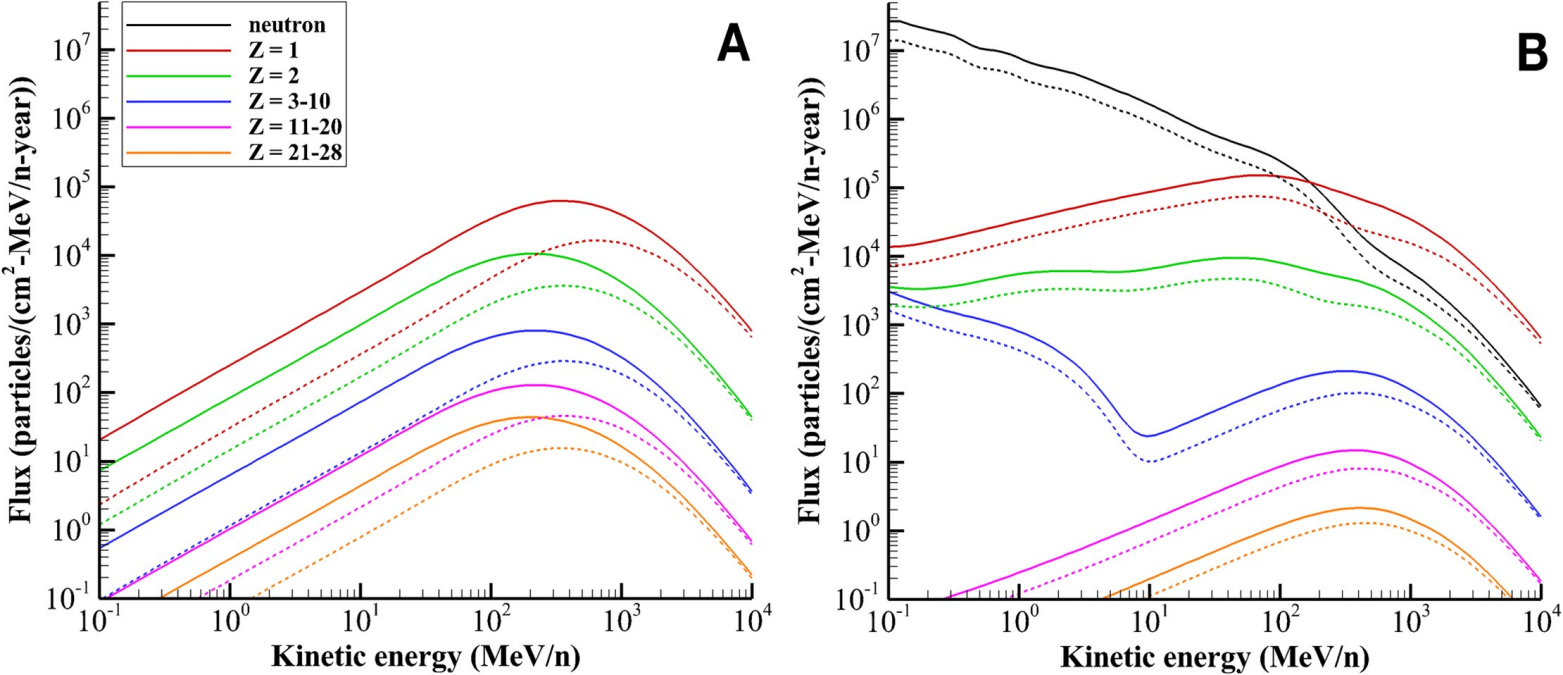
**GCR particle spectra at solar minimum conditions (June 1976) denoted by solid lines and solar maximum conditions (June 2001) denoted by dashed lines in (A) free space and (B) behind 20 g/cm**^**2**^ **of aluminum to female BFOs as described by the Badhwar–O’Neill 2010 GCR model [14], HZETRN transport code [13,16,17], and human phantoms [15,18,19].** Plot data available in S1 Data. BFO, blood-forming organ; GCR, galactic cosmic radiation; HZETRN, High Charge and Energy Transport.

The free-space GCR environment is modified as it passes through spacecraft materials as a result of atomic and nuclear interactions producing an internal environment comprised of both primary and secondary radiation, including energetic neutrons, protons, helium ions, and other nuclear fragments (Z ≥ 3). Although heavy-ion contributions to dose and dose equivalent in free space and behind light shields (on the order of 5 g/cm^2^) are large (Fig 2), behind typical spacecraft shielding, dose and dose equivalent are dominated by protons and light ions [10,12]. Further attenuating the radiation field is an astronaut’s body self-shielding. Average spacecraft shielding is on the order of 20 g/cm^2^ [10], whereas the human body has an average tissue thickness of approximately 30 g/cm^2^ for internal organ locations [15]. The modulation of the free-space environment at these combined thicknesses can be seen in Fig 3. The GCR simulator is designed to approximate this mixed field of primary and secondary particles seen at critical body organ and tissue locations within an astronaut in a shielded vehicle. In comparison with the high energies of GCR in the 10s of GeV/n, NSRL’s upper energy limit of 1.5 to 2.5 GeV/n will be the largest constraint on implementation strategy.

#### Mission doses

Over the last decades, there have been numerous studies estimating radiation exposures to astronauts from both SPEs and GCR during solar minimum and maximum conditions. These studies have assumed a wide variety of vehicle and shielding configurations with various levels of fidelity in design and material characterization, mission durations, and solar conditions [20,21,22]. Additionally, environmental models, transport codes, nuclear models, and human voxel phantoms have greatly improved our ability to more accurately project exposures [9]. Important environmental measures on both the International Space Station (ISS) and by NASA’s Mars Science Laboratory’s Radiation Assessment Detector (MSL-RAD) have provided important benchmark data to understand where models may have sometimes underestimated projected exposures [23,24]. The following estimates of mission exposures have been compiled to guide radiobiology experiments at NSRL and are largely representative of solar minimum conditions (when GCR flux is at its maximum). The range of the values in the Table 1 is representative of numerous assumptions and caveats of the individual historically based study details, as well as efforts to maintain direct comparison with environmental measurements where possible.

**Table 1:** Summary of exploration mission exposures.

Exploration Mission	Mission Duration	Dose (mGy)	Gray Equivalent (mGy-Eq)^a^	Dose Equivalent (mSv)^b^
ISS in LEO	6 months	30–60	–	50–100
ISS in LEO	1 year	60–120	–	100–200
Sortie to Gateway (free space)	30 days	20	35	55
Lunar Surface Mission (2 weeks on surface)	42 days	25	45	70
Sustained Lunar Operations	1 year	100–120	180–220	300–400
Deep-Space Habitat	1 year	175–220	300–400	500–650
Mars Mission	650 to 920 days	300–450	550–800	870–1,200

^a^Conversion of dose to gray equivalent uses RBE values recommended by NCRP No. 132 [25]

^b^Both NASA-defined quality factors [26] and ICRP 60 quality factors [11] considered in range of estimates.

**Abbreviations:** ICRP, International Commission on Radiological Protection; ISS, International Space Station; LEO, low Earth orbit; NCRP, National Council on Radiation Protection; RBE, relative biological effectiveness

ISS mission exposures are consistent with on-board and personal dosimetry measurements that vary with altitude and time in solar cycle [27]. The ISS LEO radiation environment is not simulated at NSRL and is shown here for comparison only. Mars mission exposures for conjunction class short stays (620 days free space; 30 days surface) and opposition class long stays (420 days free space; 500 days surface) are very similar [20,22,24] with estimated mission values consistent with MSL-RAD measurements. MSL-RAD measurements were 0.48 mGy/day and 1.84 mSv/day in transit and 0.20 mGy/day and 0.7 mSv/day on the Mars surface [28]. Both mission calculations provide an estimate of approximately 300 mGy and between approximately 1,120 to 1,160 mSv.

Additional analyses were performed here, specific to Mars missions (conjunction and opposition class) exposures within shielded habitats, to estimate the contribution to dose (300–450 mGy) from particle types: approximately 66% of the dose is from protons, approximately 14% to 16% from helium, approximately 4% to 5% from particles of 3 ≤ Z ≤ 9, approximately 2% to 3% from particles of Z ≥ 10, and approximately 10% to 15% from neutrons, pion, muon, and electromagnetic (π/EM) components. The range of larger contributions from neutrons and π/EM (15%) are attributed to the opposition class Mars mission with long stays on the surface resulting in increased exposure to secondaries produced in the thin Martian atmosphere and terrestrial surface.

Considering the mission exposures in Table 1, relevant GCR doses are 125, 250, 500, and 750 mGy for radiobiology experiments at the NSRL. These doses span the exploration missions under consideration by NASA used for assessing risk posture and the need for countermeasure development. A higher exposure of 750 mGy is included to establish a dose response curve above expected mission exposures supporting the evaluation of mixed-field quality effects to inform PELs. In some cases in which clinically significant endpoints are difficult to discern in animal model systems at lower doses, such as the determination of quality effects on the cardiovascular system, exposures up to 1.5 Gy may be warranted.

#### Comparison of implementation strategies

**Fig 4:**
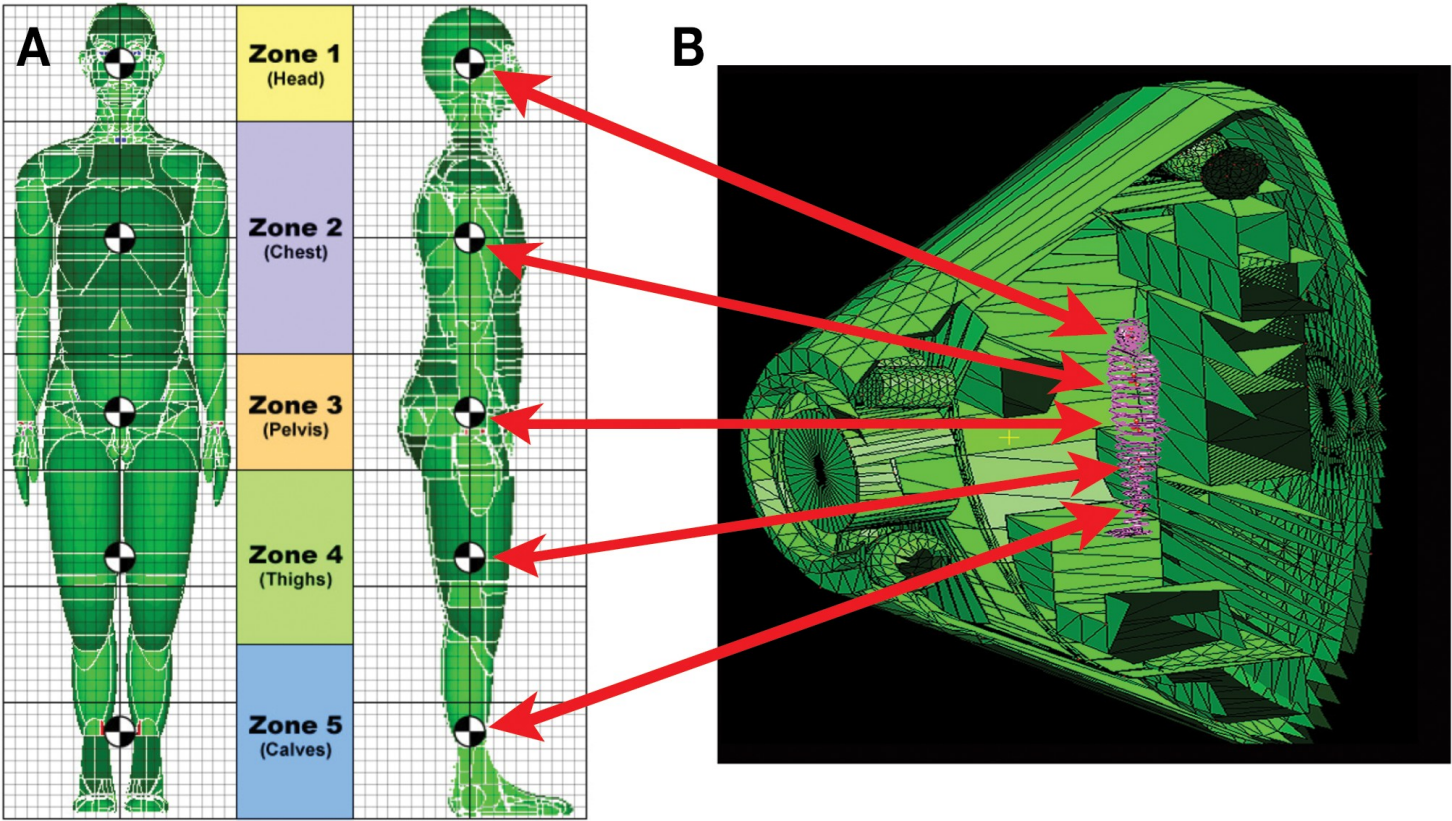
Vehicle shielding is combined with shielding afforded by a crew member’s body surrounding critical organs to determine the primary and secondary radiation environment at points within the crew member. (A) Human phantoms are used to calculate the body’s self-shielding of critical organs. (B) Shield thickness provided by the vehicle are depicted as green intersecting rays in a crew exploration vehicle (similar to Orion).

The simulator is designed to expose animal models and cell culture systems to the environment seen by crew at critical body organ and tissue locations within shielded vehicles. This is illustrated in Fig 4 where the external GCR environment is transported through the vehicle shield geometry and then through the self-shielding provided by the body. For example, the particle spectrum seen at a point within the body of an astronaut is the reference particle spectrum to be delivered to the biological sample. As described later, the self-shielding provided by the mouse or rat will become important for range considerations of ions selected to ensure a nearly uniform dose distribution within the animal.

**Fig 5:**
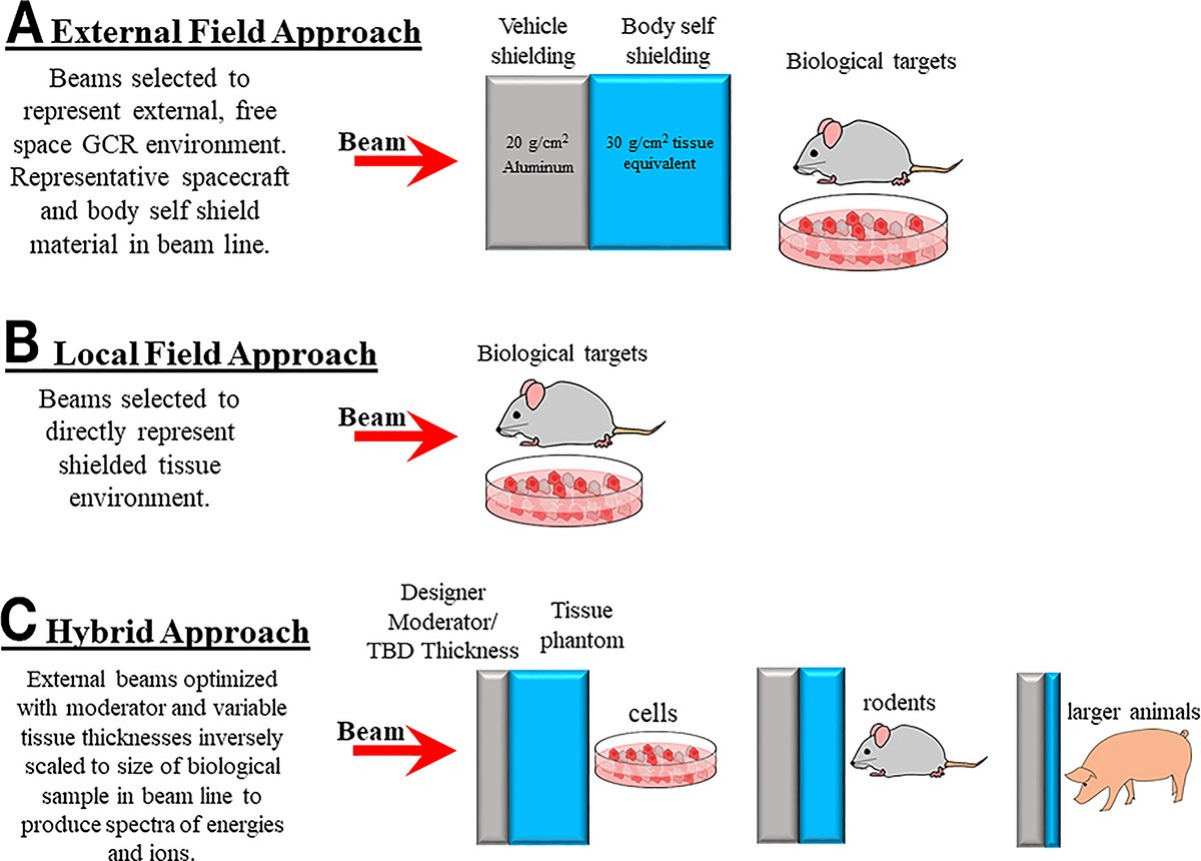
Three basic strategies for beam selection. (A) Beam selection is representative of the external, free-space GCR spectrum and is approximated by discrete ion and energy beams delivered onto a shielding and tissue equivalent material placed within the beam line, in front of the biological target. (B) Beam selection is representative of the shielded tissue spectrum found in space (e.g., average tissue flux behind vehicle shielding) and is approximated by discrete ion and energy beams delivered directly onto the biological target. (C) Beam selection is representative of energies less than free space with thinner amounts of vehicle shielding and variable thicknesses of tissue equivalent materials to represent the differences in body self-shielding between the physical sizes of species. GCR, galactic cosmic radiation.

Three basic implementation strategies were considered (Fig 5): an external field approach, a local tissue field approach, and a hybrid approach. In the external field approach, discrete NSRL beams are selected to represent the free-space, external GCR field, and the biological target is placed behind shielding materials in the beam line. The shielding material is sized to modify the primary beams in a manner similar to spacecraft and body-self (tissue) shielding. In the local tissue field approach, models are used to characterize the spectrum of particles and energies occurring in critical body organs behind shielding. This modified spectrum is then represented by the accelerator with discrete monoenergetic beams that are delivered directly to the biological target with no intervening shield material in the beamline. The hybrid approach considers an optimum amount of shielding within the beamline with variable tissue equivalent material thicknesses. The goal of this approach is to generate secondary particles with a spread in energy and charge and to allow for correlated secondary ion interactions within cells. The tissue equivalent shielding also provides a moderator that may be scaled to account for the different physical sizes of animal models. In this case, the external field (of higher energies than local tissue approach) delivered by the accelerator and simulated vehicle and tissue shielding (less shielding than used with the external field approach) would be modified to best represent the particle spectrum seen within an astronaut.

The first 2 approaches were thoroughly analyzed by Slaba and colleagues, 2016 [8], considering simple shield geometries of 5, 10, and 20 g/cm^2^. Analysis showed that the NSRL upper energy constraints limit the feasibility of simulating the external, free-space GCR spectrum directly. In particular, it was shown that approximately half of the exposure behind shielding is lost if the energies above approximately 1.5 GeV/n cannot be represented in the external field. However, the shielded, local tissue environment can be reasonably well represented, with approximately 85% of effective dose captured, within current facility capabilities with energies of protons up to 2.5 GeV, helium up to 1.5 GeV/n, and heavier ions up to 1.5 GeV/n. To achieve this same effectiveness for the external field approach, upper energy limits would need to be increased to approximately 5 GeV/n to 8 GeV/n depending on estimated shield thicknesses used in the beamline. Thus, the GCR simulator is designed to approximate a reference environment based on the local tissue approach to best describe mission exposures encountered by the crew (Fig 5B). Future work will consider the advantages and disadvantages of a hybrid approach.

### Determination of GCR reference environment

#### Impact of mission parameters

Radiation exposure to the crew is mission specific and dependent on multiple factors such as mission destination and duration, vehicle design, and solar conditions. The impact of these modifiers on establishing a single GCR reference field is summarized here based on the work of Slaba and colleagues, 2016 [8]. A series of computational tools and models were used in the analysis including the Badhwar–O’Neill GCR environment model [14], the High Charge and Energy Transport (HZETRN) transport code [13,16,17], vehicle computer-aided design (CAD) geometries, and human phantoms [15,18,19]. A complete discussion of the computational tools and models supporting analyses such as these can be found in the review article by Norbury and colleagues, 2019 [9]. The methodologies integrating these analysis tools have been described by Singleterry and colleagues, 2011 [29], and are available on NASA’s radiation design tool website, On-Line Tool for the Assessment of Radiation in Space (OLTARIS; https://oltaris.nasa.gov/).

Various physical quantities and associated uncertainties were assessed to determine the best way to quantify reference field parameters. The modification of the free-space GCR environment through a broad range of shielding configurations, including complex vehicles and habitats (e.g., models of the ISS and Space Transportation System) and simplified spherical shielding of 5, 20, and 40 g/cm^2^ aluminum, were considered to quantify variability in the induced tissue field. Variation in dose (Gy) across all major radiosensitive tissues, including the heart and brain, were assessed. The variation across all tissues and geometries was found to be ±3% for dose and ±16% for dose equivalent. Dose equivalent (Sv) is slightly more sensitive than dose to shielding thickness due to the added emphasis placed on HZE ions by the quality factor. Although dose equivalent is specific to carcinogenesis, it was deemed an important quantity to consider in the design to verify that certain characteristics (i.e., HZE interactions) of the reference field are maintained by the beam selection.

Similar comparisons can be made in assessing the modified GCR spectrum in terms of flux versus linear energy transfer (LET). LET is an important quantity describing the average amount of energy deposited by ionizing radiation per unit length, usually expressed in keV/μm, and is related to a particle’s charge and velocity (energy per nucleon) and the elemental composition of the traversing medium (or tissue in this case). The tissue LET spectra behind various complex and simple shield geometries and at critical organ locations also suggest little variation in important regions. Differences were clearly seen at LET values greater than 200 keV/μm; however, the flux of particles with such high LET is quite small, and therefore, the region does not contribute heavily to dose or dose equivalent. Below 10 keV/μm, a region that contributes heavily to dose and dose equivalent, spectral results were nearly indistinguishable. It was found that 20 g/cm^2^ of spherical aluminum shielding adequately represented the suite of geometries considered, and the blood-forming organ (BFO) results were found to be near the average of all critical tissue exposures. The trend for BFO is attributed to the distributed nature of the tissue sites found throughout the body.

The impact of solar activity was assessed, and as expected, the magnitude of the tissue exposures was found to change substantially between solar minimum and solar maximum. However, it was shown that this variation can be approximately represented by a single scale factor of 1.85, which corrected differences in tissue exposures associated with solar activity to within ±7% over the range of shielding configurations. Differential LET spectra in tissue behind shielding for solar minimum and solar maximum were also shown to be very similar across the entire LET spectrum using the single scale factor. This indicates that the spectral characteristics of the radiation field encountered by shielded astronauts during solar minimum and maximum are qualitatively comparable for GCR simulator design, and only the total dose and dose rate of the reference field would need to be modified to account for the impact of solar cycle.

Through the analysis of Slaba and colleagues, 2016 [8], the reference field was chosen as the female BFO spectrum behind 20 g/cm^2^ of aluminum shielding during solar minimum conditions. It was found that variation in dose, dose equivalent, and LET spectrum introduced by shielding geometry and identification of the BFO to represent critical organ and tissue exposures is likely small compared with uncertainties associated with facility limitations in representing the full reference field by a discrete number of monoenergetic beams. Other quantities, such as dose equivalent and the track structure parameters [26], were used to independently verify that certain characteristics of the reference field are maintained by the beam selection as described by Slaba and colleagues, 2016 [8], but are not discussed here. The tissue energy and LET spectra behind shielding and calculated dose per particle type will be used to guide NSRL beam delivery requirements.

#### Definition of reference field quantities

Characteristics of the reference field are shown in Fig 3 comparing the free-space GCR ion spectral flux as a function of energy to the attenuated spectrum within tissue. The build-up of secondary protons, light ions, and neutrons can be seen compared to the attenuation of heavy ions. In the BFO behind 20 g/cm^2^, the *Z* = 1 ions account for approximately 64% of dose, the *Z* = 2 ions account for approximately 17% of dose, and ions of Z ≥ 3 contribute approximately 7% of dose as shown in Table 2. The analysis of Slaba and colleagues, 2016 [8], showed that the HZE particles with the largest contribution to dose in the reference field were: C, N, O, Ne, Mg, Si, Ca, and Fe, which make up 69% of the HZE dose. This is not surprising, considering the relative abundance of HZE particles (Fig 2) in the natural GCR spectrum. It is important to recognize and clarify how the contributions of secondary neutrons to dose, through elastic and inelastic collisions occurring within tissue, are evaluated. The radiation transport code, HZETRN [13,16,17], used to calculate reference field quantities, propagates secondary neutrons and any of their reaction products with charge of Z ≤ 2. Of particular interest are the recoil protons generated within tissue by mainly low-energy neutrons. These protons are explicitly included in the Z = 1 component of the reference environment and are well represented in the GCR simulator by the binary filter approach (See Beam selection) implemented for protons with energy < 100 MeV. Heavy target fragments (Z ≥ 3) produced from inelastic neutron interactions, with approximately 0.8% dose contribution, are not explicitly included in the reference environment. These neutron interaction products will contribute more significantly (approximately 10%) to biologically weighted exposure quantities such as dose equivalent or risk of exposure-induced death (REID). In the local field approach, these particles are not directly simulated in the NSRL beam line. Likewise, π/EM cascades, which contribute approximately 11% to dose, are also not explicitly represented in the GCR simulator. Contributions to dose are normalized in Table 2, excluding the contribution of these 2 components of the spectrum. Thus, the GCR simulator beam selection is designed to deliver the majority of the dose from hydrogen (approximately 65%–75%) and helium ions (approximately 10%–20%) with heavier ions (Z ≥ 3) contributing the remainder (6%–8%). As previously discussed, these results are consistent with model calculations and measurements for Mars mission exposures [10,12].

**Table 2:** Average tracks per cell nucleus per year, dose (mGy/year), and percent contribution of particles to dose for reference field during 1-year solar minimum and normalized to 500 mGy.

Particle Type	Average tracks per cell nuclei^a^	Dose (mGy/Year)	Percent contribution (%)	Dose distribution normalized to 500 mGy	Percent contribution of normalized dose to 500 mGy (%)
π/EM	0.1	15.5	11.6	0	0
Neutron	N/A	1.1^b^	0.8	0	0
hydrogen	126	86^c^	64.2	366.3	73.3
helium	7	22.5^d^	16.8	95.8	19.2
HZE	0.5	8.9	6.6	37.9	7.6
total	133.6	134	100	500	100

^a^Assumes a cell nucleus cross section of 100 μm^2^

^b^Dose exclusively from heavy target fragments (Z ≥ 3) produced from inelastic neutron interactions.

^c^Includes contributions from elastic and inelastic reaction products of Z = 1 from neutron interactions.

^d^Includes contributions from inelastic reaction products of Z = 2 from neutron interactions.

**Abbreviations:** EM, electromagnetic radiation; HZE, high charge and high energy ions

**Fig 6:**
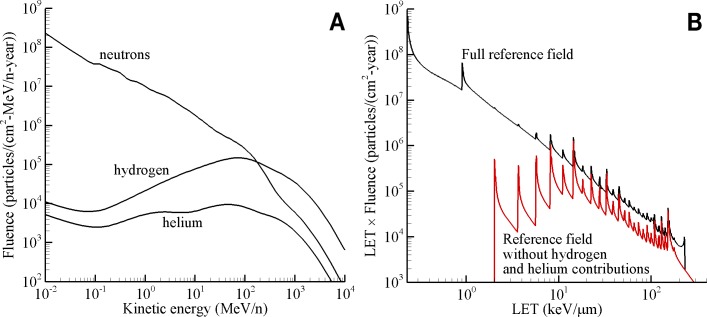
Reference field spectra in the female BFOs behind 20 g/cm^2^ of aluminum shielding during solar minimum conditions. (A) Neutron, hydrogen, and helium energy spectra. (B) The corresponding differential LET spectra with and without contributions from hydrogen and helium. Based on calculations from Slaba and colleagues, 2016 [8]. Plot data available in S1 Data. BFO, blood forming organ; LET, lineal energy transfer.

The reference field spectral quantities and integral quantities provide a means to optimize the selection of beam parameters. The neutron, hydrogen, and helium energy spectra from the reference field are shown in Fig 6A. The hydrogen spectrum includes protons (^1^H), deuterons (^2^H), and tritons (^3^H), and the helium spectrum includes helions (^3^He) and alphas (^4^He). These spectra explicitly include elastic and inelastic reactions products from neutron interactions. Fig 6B shows the total differential LET spectrum of the reference field and the differential LET spectrum without the hydrogen and helium contributions. The numerous peaks appearing in the plot are associated with singularities introduced when transforming flux as a function of kinetic energy into flux as a function of LET. The location of the peaks along the horizontal axis are characteristic of individual ions. The quantity plotted on the vertical axis, fluence, has also been scaled by LET to improve plot clarity. Integrating under the curves shown in Fig 6 yields the total annual dose (standard constants to convert from MeV/g to Gy would need to be applied) of 134 mGy (shown in Table 2). The tissue energy spectra of protons and helium particles and the tissue LET spectrum of heavier charged particles of (Z ≥ 3) behind shielding are used to guide the selection of NSRL beams.

### Implementing the GCR simulator at the NSRL

In early 2017, the GCR simulator reference field (Fig 6) was baselined by NASA for implementation at the NSRL. A single reference field was defined to ease requirements on facility operations, to enhance cross comparison of radiobiological results between PI teams, and to increase return on investment for secondary science objectives through tissue sharing. A general beam selection strategy to best simulate the field was developed in consultation with BNL’s Collider Accelerator Department, who are responsible for the delivery of beams to the NSRL target room. Although the primary and secondary field of GCR is comprised of a spectrum of many ions over a broad energy range, early simulation at NSRL was limited to relatively few monoenergetic beams to maintain reliability and repeatability.

#### Beam selection

**Fig 7:**
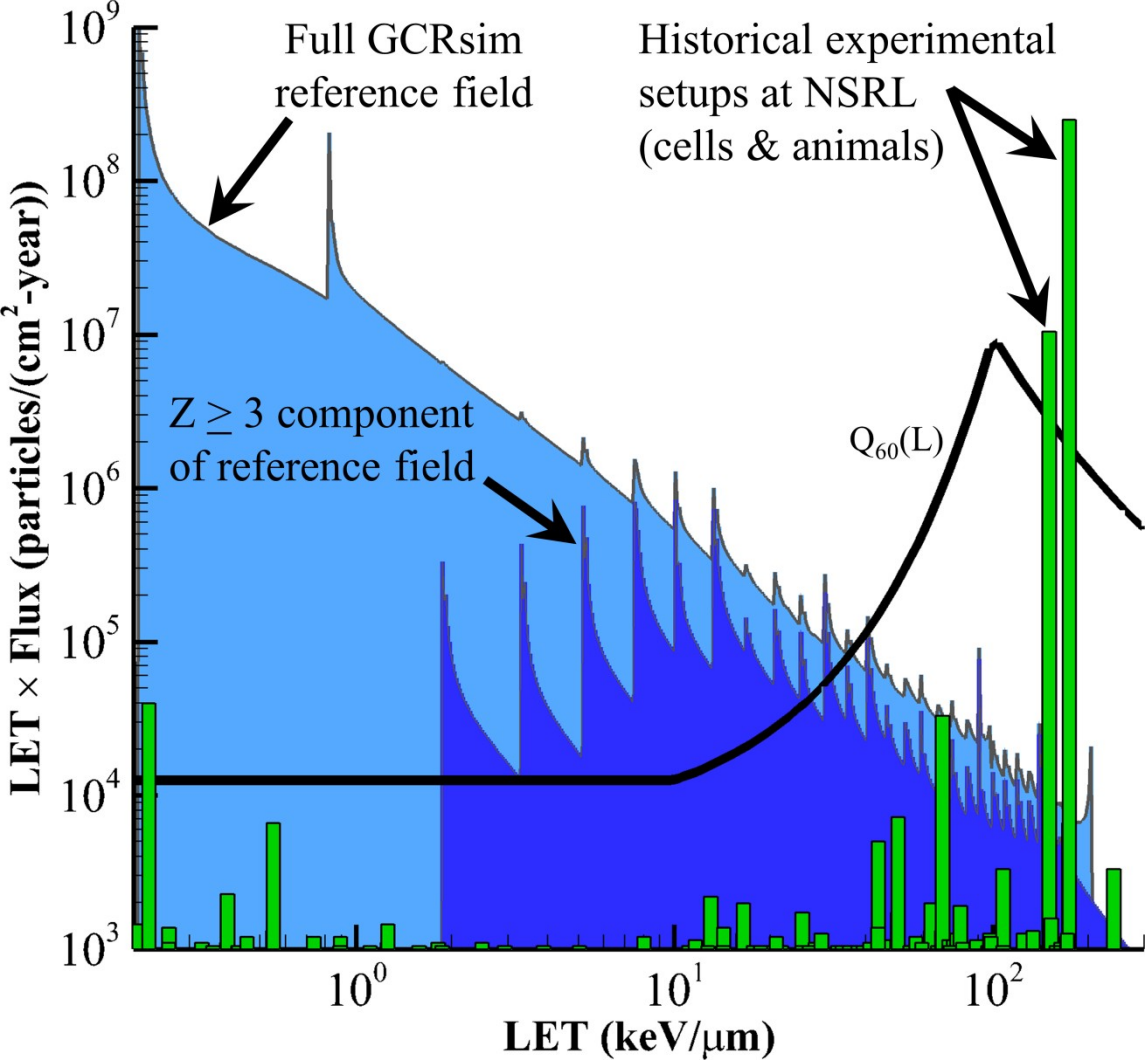
Illustration of beam selection strategy for GCR simulator. The total LET spectrum (light blue) and the HZE spectrum (dark blue) are shown separately. The green bars are representative of the number of single-ion beam experiments performed at NSRL as a function of LET (scaled for plot clarity). The black line is representative of ICRP-60 quality factor weighting [11] to estimate biological damage (scaled for plot clarity). Plot data available in S1 Data. GCR, galactic cosmic radiation; HZE, high charge and high energy ions; ICRP, International Commission on Radiological Protection; LET, linear energy transfer; NSRL, NASA Space Radiation Laboratory.

Several key decisions were made in efforts to define and demonstrate an early baseline capability to accelerate NASA research objectives. First, the reference spectrum was defined in terms of the physical quantities of flux versus energy and flux versus LET as described in the previous section. The Z = 1, Z = 2, and Z ≥ 3 portions of the reference field were considered individually. Beam selection favored those ions with a wealth of historical radiobiological data, ions that provided LET and energy coverage of the reference field, as well as consideration of their contribution to biological damage relative to the International Commission on Radiological Protection (ICRP) quality factor weighting [11]. In addition to beam energy, the number of discrete monoenergetic ions was thought to be limited by the production capability within the Electron Beam Ionization Source (EBIS) to 6 to 8 ions with no more than 2 gases being used as sources. The general scheme is notionally illustrated in Fig 7.

**Fig 8:**
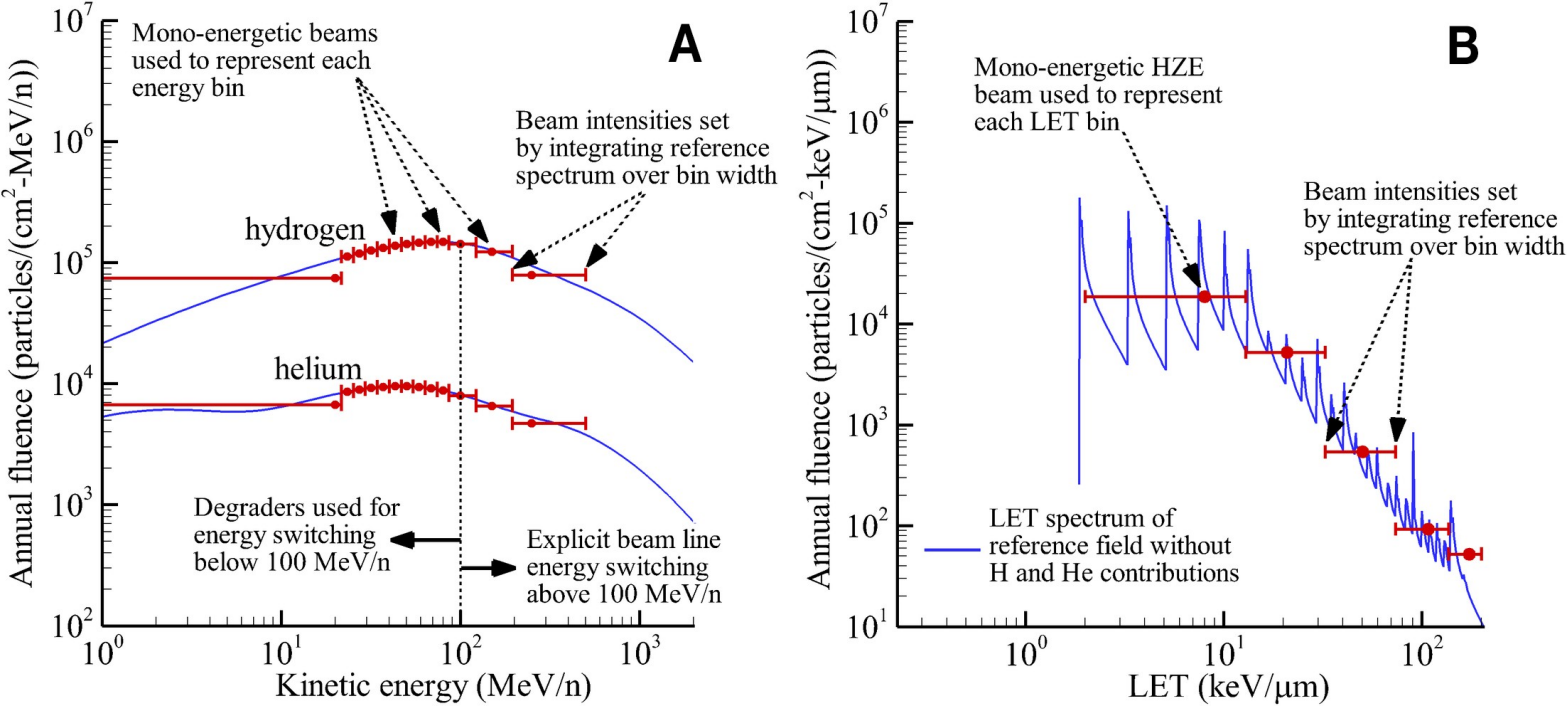
Representation of the reference field using discrete monoenergetic beams. The hydrogen and helium energy spectra are considered directly (A), whereas HZE ions are represented within the LET spectrum (B). Solid blue lines are the reference spectra from Fig 6. The bin widths for 1 GeV/n protons and helium particles are at lower fluences and not shown on the figure; however, these data are included in supplementary data file. All plot data available in S1 Data. HZE, high charge and high energy ions; LET, linear energy transfer.

From an historical perspective at NSRL, the most used ions (as represented by the green bars on Fig 7) include iron (1,000 MeV/n, 600 MeV/n), protons (1,000 MeV/n, 150 MeV/n), silicon (600 MeV/n, 300 MeV/n), with fewer runs of titanium (1,000 MeV/n), oxygen (600 MeV/n, 250 MeV/n), and carbon (290 MeV). Given that hydrogen and helium account for a large fraction of overall fluence and dose over a broad energy range, these particle are given the greatest emphasis in beam selection and are handled individually. Monoenergetic particles are selected to represent the physical quantity of flux over a specific energy (MeV) range. This separates the contributions of protons from helium in regions where LET values overlap. The NSRL’s binary filter will be used to generate a “continuous” low-energy spectrum of proton and helium particles below 100 MeV/n. The binary filter is a variable thickness degrader system made of polyethylene (density of 0.93 g/cm^3^) sheets that can be remotely inserted into the beamline to slow down or stop incoming particles (see NSRL facility modifications). Although NSRL can generate lower energy protons and alphas down to 50 MeV/n, utilizing the binary filter allows for more efficient operations, requiring a fewer number of discrete beam energies (less energy switching) and provides a reasonably smooth dose distribution (similar to approximating a spread-out Bragg peak) within the biological target. A smaller number of specific monoenergetic HZE beams are selected to collectively represent the associated HZE or high-LET portion of the reference spectrum in which multiple ions may contribute within a range of LET values. The implementation of this strategy is shown in Fig 8.

Selection of discrete proton and alpha beams included 1,000 MeV/n and 250 MeV/n based on historical data and the additional beams of 150 MeV/n and 100 MeV/n to capture energy range of interest. It was decided to use monoenergetic beams of protons and alphas of the same energies (MeV/n). To establish the proton and alpha intensities to be delivered by the beam, discrete energy bins were assigned to each of the monoenergetic ions with the corresponding intensity determined by integrating the reference field over the indicated energy bin to obtain total fluences. Below 100 MeV/n, discrete proton and alpha beam energies are achieved by using the polyethylene degrader system. Ten log-spaced energy bins were defined between 20 MeV/n and 100 MeV/n. It was recognized that protons and alphas below 20 MeV/n would not have sufficient energy to penetrate through the animal exposure boxes (approximately 2 mm of polyethylene) and reach radiosensitive organs. Therefore, 20 MeV/n was the lowest beam energy used and the lower bin limit was set to 0 MeV/n for integration to capture the total fluence of protons and alphas contributing to reference field integral quantities. As will be seen, the entrance dose of the 20 MeV/n beams may appear artificially high compared with the next highest energy but will contribute negligibly to internal exposures and biological outcomes. Above 100 MeV/n, proton and alpha beams are delivered directly by the accelerator at the 4 selected energies. The proton and alpha energy bin limits above 100 MeV/n are determined by considering the geometric mean of adjacent energies. To establish the upper bin width for integration represented by the 1 GeV/n beams, the upper energy limit was set at 50 GeV/n for both protons and alphas.

For ions of Z ≥ 3, the LET domain is separated into 5 discrete, log-spaced bins as shown in Fig 8B. Based on historical data and requirement to cover the range of LET values represented by ions with Z ≥ 3, the following HZE beams were selected for the reference field: ^12^C (1,000 MeV/n) and ^16^O (350 MeV/n) were selected to be representative of particles of 3 ≤ Z ≤ 9; and ^28^Si (600 MeV/n), ^48^Ti (1,000 MeV/n), and ^56^Fe (600 MeV/n) were selected to be representative of particles of Z ≥ 10. To set the HZE beam intensities, discrete LET bins were assigned to each of the 5 monoenergetic ions with the corresponding intensity determined by integrating the reference field over the indicated LET bin to obtain total fluences. For carbon, the lower bin limit is set to 2 keV/μm, which corresponds to the lowest LET for Z = 3 ions in water. The upper bin limit is set as the geometric mean of the carbon and the adjacent oxygen LET value (found to be 12.9 keV/μm). Bin limits for oxygen, silicon, and titanium are similarly defined by considering the geometric mean of LET values for adjacent beams. For iron, the upper LET bin limit was selected as 200 keV/μm to capture the region important to biological systems beyond which RBE continues to decrease as approximately 1/LET^0.5^.

To further aid the development of the GCR simulator, NASA established a “GCR Experimental Consortium” in 2015 to enhance the outcomes from early mixed-field experiments supporting updates to the NSRL implementation of the GCR reference field. Experimental endpoints, mixed-beams, and doses were aligned across 3 PI-led research teams with the goal of leveraging large historical data sets of single-ion experiments to develop and validate predictive models (including theories of simple additivity, synergy, or antagonistic effects of multiple ion exposures) across multiple endpoints. Given historical data and the alignment of experimental designs, the following beams were identified by NASA and the consortium: protons (1,000 MeV/n, 250 MeV/n), helium (250 MeV/n), oxygen (350 MeV/n), silicon (263 or 330 MeV/n), titanium (300 MeV/n), and iron (600 MeV/n). Efforts were made to match experimental consortium beams (ion species and energies) with those beams under consideration for the baseline GCR simulator capability. Published results will inform our understanding of the sensitivity of biological response to the exact composition of the selected mixed-field particle species, to the order of ion delivery, to the possibility of synergy among components of the mixed GCR, and to the timing of beam delivery. Early results investigating synergy between various GCR ion beams on the prevalence of murine Harderian gland tumorigenesis have been published [30], which illustrate the importance of considering the physics of space radiation interactions in tissue, such as the information presented here in the development of the simulator, to interpret biological responses to mixed-field exposures and ultimately NASA GCR simulations.

Following the beam selection strategy discussed above, the reference field is specifically defined by the ion-energy beam combinations shown in Table 3. The sequentially delivered mixed field consists of 33 beams including 4 proton energies plus degrader, 4 helium energies plus degrader, and the 5 heavy ions of ^12^C (1,000 MeV/n), ^16^O (350 MeV/n), ^28^Si (600 MeV/n), ^48^Ti (1,000 MeV/n), and ^56^Fe (600 MeV/n). A binary polyethylene filter is used with the 100 MeV/n H and He beams to provide the distribution of low-energy particles between 80 and 20 MeV/n. The NSRL GCR simulator reference field is normalized to a Mars mission relevant exposure of 500 mGy as shown in Table 3. Other doses of interest are 125, 250, and 750 mGy, depending on animal model, endpoints, and animal numbers required for statistical significance. These doses are implemented as fractions or multiples of Table 3. Initial research studies have used 500 mGy.

**Table 3:** “NSRL GCR Simulation” beam definition normalized to 500 mGy.

**Primary ion-energy beam combinations in GCR simulator**				**Dose** **(mGy)**	**Fractionated dose- 24 exposures** **(mGy/day)**
**Ion**	**E (MeV/n)**	**LET (keV/ μm)**	**Range (cm)**		
^1^H	20–100	Polyethylene degrader to lower energies		140.6	5.86
^1^H	150	0.54	15.9	35	1.46
^1^H	250	0.39	38.1	68.9	2.87
^1^H	1,000	0.22	326.6	123.6	5.15
^4^He	20–100	Polyethylene degrader to lower energies		39.6	1.65
^4^He	150	2.17	16	7.5	0.31
^4^He	250	1.56	38.3	16.4	0.68
^4^He	1,000	0.88	327.8	24.9	1.04
^12^C	1,000	7.95	110.13	11.7	0.49
^16^O	350	20.8	16.95	15.4	0.64
^28^Si	600	50.2	22.73	8.1	0.34
^48^Ti	1,000	109.5	32.53	4.5	0.19
^56^Fe	600	175.1	13.09	4.1	0.17
			Total	500	20.8
Ten lower energy proton beams from 100 MeV/n proton incident on degrader system					
**Ion**	**E (MeV/n)**	**LET (keV/ μm)**	**Range (cm)**	**Dose** **(mGy)**	**Fractionated dose- 24 exposures** **(mGy/day)**
^1^H	20	2.59	0.43	30.4	1.3
^1^H	23.3	2.29	0.56	6.7	0.3
^1^H	27.2	2.02	0.75	7.4	0.3
^1^H	31.7	1.79	0.98	8	0.3
^1^H	37	1.58	1.3	8.7	0.4
^1^H	43.2	1.39	1.72	9.3	0.4
^1^H	50.3	1.23	2.26	10	0.4
^1^H	58.7	1.09	2.99	10.6	0.4
^1^H	68.5	0.97	3.95	11.1	0.5
^1^H	79.9	0.86	5.2	11.2	0.5
^1^H	100	0.73	7.76	27.2	1.1
Ten lower energy helium beams from 100 MeV/n helium particle incident on degrader system					
**Ion**	**E (MeV/n)**	**LET (keV/ μm)**	**Range (cm)**	**Dose** **(mGy)**	**Fractionated dose- 24 exposures** **(mGy/day)**
^4^He	20	10.34	0.43	11	0.5
^4^He	23.3	9.14	0.57	2.1	0.1
^4^He	27.2	8.06	0.75	2.2	0.1
^4^He	31.7	7.12	0.99	2.3	0.1
^4^He	37	6.29	1.31	2.5	0.1
^4^He	43.2	5.56	1.73	2.6	0.1
^4^He	50.3	4.92	2.28	2.7	0.1
^4^He	58.7	4.36	3.01	2.7	0.1
^4^He	68.5	3.86	3.97	2.7	0.1
^4^He	79.9	3.43	5.23	2.7	0.1
^4^He	100	2.9	7.81	6.1	0.3

A number of radiobiology studies have indicated that the order of exposure to protons and heavy ions may be an important parameter for GCR simulator design due to varied responses observed for different sequences of fractionated doses [31,32]. Given estimates of average hits per cell exposed to the shielded reference field (Table 2), each human cell will be traversed by a proton approximately 100 times/yr, by a helium ion approximately 6 times/yr, and by an HZE ion approximately 0.5 times/yr. Thus, most cells in astronauts will be hit by a proton(s) before being hit by a helium or HZE ion. The ordering of the particles in the simulator has taken this into account with protons and then helium beams delivered prior to heavy-ion beams to approach space-like conditions.

A second consideration in the ordering of beams was to minimize the number of unique beams (ion and energy switches) delivered to the NSRL from the Booster synchrotron. To ease operations, 2 low-energy protons are grouped and 2 low-energy helium ions are grouped sequentially throughout the delivery sequence so that only changes in the binary filter system are required. For example, a 100 MeV/n proton beam generates both the 20 MeV/n and 23 MeV/n beams with use of a degrader in the NSRL beamline. Lastly, in efforts to simulate a continuous (or highly sequential) background of protons with some helium and sporadic heavy ions, heavy ion beams are seperated by proton and helium beams. The GCR simulator supplies the beams in Table 3 in the following order:

- (H 1,000*), (He 1,000), (Si 600)
- (H 20), (H 23), (He 20), (He 23), (Ti 1,000), (He 27), (He 32), (H 27), (H 32)
- (H 37), (H 43), (He 37), (He 43), (O 350), (He 50), (He 58), (H 50), (H 58)
- (H 68), (H 80), (He 68), (He 80), (C 1,000), (He 100), (H 100)
- (H 150), (He 150), (Fe 600), (He 250), (H 250)

*denotes energy in MeV/n

This sequence requires 21 switches of unique ion-energy beams to the NSRL.

#### Analysis of dose distribution within animal models

The GCR simulator is designed to expose biological targets, such as mice and rats, to the internal radiation environment seen at critical organ locations within the human body as depicted in Fig 4. Additionally, NASA research studies require that animal model systems are reflective of the age of astronauts which is 35 to 55 years old. Several upcoming GCR simulator studies are planning to use socially mature Wistar rats that are 5 to 9 months old and weigh approximately 700 to 800 g. Given that some of the selected low-energy proton and helium beams have short ranges, there is concern that these beams may stop in the animal creating a sharp dose distribution (i.e., hot spots) with the possibility of highly localized tissue responses. Additional transport studies using phantom mouse and rat models have been completed to ensure that the selected GCR simulator beam energies can provide a homogeneous dose distribution within the animal’s internal organs. This explicitly now takes into account the tissue self-shielding of the rodent model system using computed tomography (CT)-based models as well as the approximately 2-mm thickness of the exposure cages (see Animal and cell handling).

**Fig 9:**
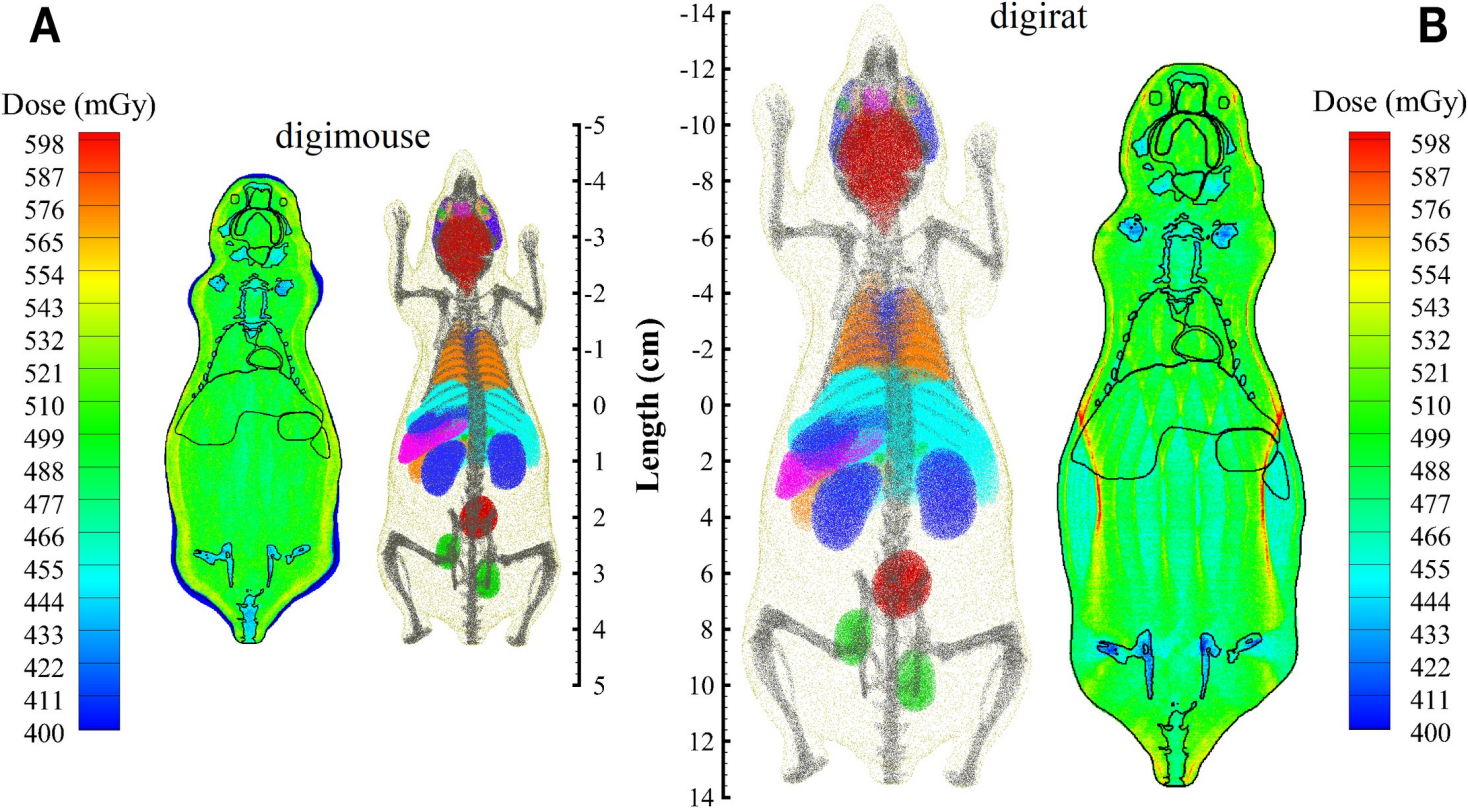
Mouse and rat voxel models used to evaluate dose distributions in tissues from exposure to GCR simulation. Digimouse (A) has been scaled by a factor of 3.15 to obtain and estimate of a rat’s body self-shielding, referred to here as “digirat” (B). Transport of full GCR simulation field provides homogeneous dose distribution within voxel mouse model (A) and scaled rat model (B). GCR, galactic cosmic radiation.

The Digimouse, a 3D mouse atlas from University of Southern California (http://neuroimage.usc.edu/neuro/Digimouse), is based on CT and cryosection images of a 28-g normal male mouse and has a model resolution (voxel dimension) of 0.1 mm (see Fig 9A). Each voxel has been segmented to identify major organs and tissues, including heart, liver, lungs, stomach, and brain. A larger “digirat” model is obtained here by directly scaling the Digimouse, resulting in voxel dimensions being increased by a factor of 3.15 with a resulting body mass of 754 g (Fig 9B). The voxel models have been coupled to the Monte Carlo transport code, Geant4 [33], for radiation transport of single-particle beams and the complete 33 ion beam GCR simulator.

The resulting dose distributions are also shown in Fig 9 for Digimouse and “digirat.” Given the cage sizes and experience from “dry runs,” a pseudoisotropic irradiation (along the 6 coordinate axes) was used for the Digimouse assessment and a lateral irradiation (perpendicular to the left and right flanks) was used for the “digirat” assessment. The average dose to the Digimouse organs was 500.7 mGy, comparing well to the externally delivered GCR simulator dose of 500 mGy. The dose distribution within the soft tissues varied by less than 3% as shown in Table 4. The average voxel dose was 513.7 mGy with 95% of the voxel doses found to be within 7% of the average dose, indicating no locally high-dose gradients. For the “digirat,” the 33 beams were oriented in a two-directional field, both laterally, on the phantom and then averaged to calculate the dose distribution. The average dose to organs was 490.7 mGy, also comparing well to the externally delivered GCR simulator dose of 500 mGy. The dose distribution within the rat soft tissues varied by less than 5% as shown in Table 5. The average voxel dose was 492 mGy with 95% of the voxel doses found to be within 10% of the average also indicating a relatively smooth dose distribution. As expected with the larger animal size and beam directions limited to 2 orientations, a slight dose gradient is seen in both outer flanks of the “digirat” compared with the mouse. However, with the natural movement of the animal within the cage, the resulting dose gradient in the flanks will be much less than calculated above, and, therefore, no locally high tissues doses or hot spots are anticipated. The “digirat,” weighing approximately 25 times more than Digimouse, assessment supports the expectations that body shielding from mouse cagemates will not interfere with delivering a consisitent dose distribution. Additional range considerations to deliver homogenous low-energy proton and helium dose distributions from the degraded spectra may be required for species larger than rats.

**Table 4:** Calculated Digimouse tissue and skeleton doses after pseudoisotropic (6 direction) irradiation with GCR simulator beams.

Tissue	Dose (mGy)	Relative diff. from average (%)
skin	507.8	1.4
skeleton	449.3	−10.3
eye	510.5	2.0
brain	510.1	1.9
heart	492.6	−1.6
bladder	492.8	−1.6
stomach	498.0	−0.5
spleen	513.5	2.6
pancreas	506.4	1.1
liver	497.6	−0.6
kidneys	504.5	0.8
lungs	494.7	1.2
Average	500.7	

**Abbreviations:** GCR, galactic cosmic radiation

**Table 5:** Calculated “digirat” tissue and skeleton doses after nonisotropic (2-direction) irradiation with GCR simulator beams.

Tissue	Dose (mGy)	Relative diff. from average (%)
skin	491.3	0.1
skeleton	438.3	−10.7
eye	493.3	0.5
brain	501.7	2.2
heart	494.1	0.7
bladder	466.5	−4.9
stomach	492.0	0.3
spleen	483.0	−1.6
pancreas	499.4	1.8
liver	488.1	−0.5
kidneys	483.6	−1.4
lungs	495.6	1.0
Average	490.7	

**Abbreviations:** GCR, galactic cosmic radiation

**Fig 10:**
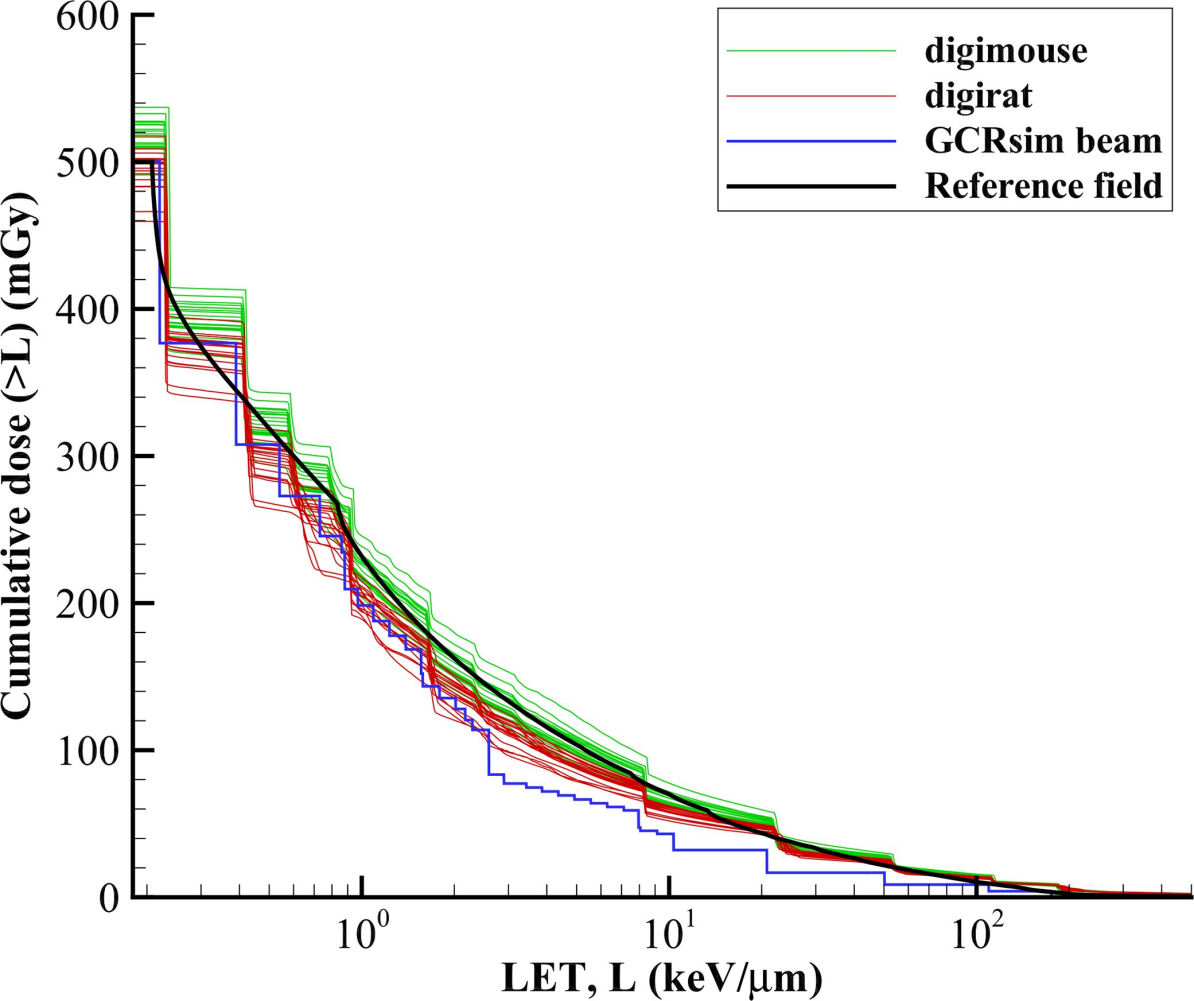
Cumulative dose as a function of LET comparing simulated environments within phantoms to the reference field and GCR simulation beam exposure. Plot data available in S1 Data. GCR, galactic cosmic radiation; LET, linear energy transfer.

The resulting cummulative dose distributions as a function of LET are compared in Fig 10 for transport of both the full reference GCR environment and the 33 GCR simulator ion-energy beam combinations of Table 3. As shown in Fig 10, good agreement is achieved. Additional analyses are underway to consider a fluence-based approach, using both the Digimouse and voxelized rat phantom, to calculate the number of voxel traversals and cell hits within a given organ for select beams. This supports simulating the GCR environment through comparison of the number of cell hits within critical body organs of a shielded crew member and approximating the same cell hits per irradiated rodent organs in the overall beam delivery strategy.

#### Dose-rate studies

Exposures from GCR consist of a complex field of many different types of particles that deposit dose at a relatively low rate. Data on dose-rate effects for higher LET radiation and more specifically dose-rate effects in a space-like environment are limited. Evidence suggests that biological responses seen at high dose rates may be different than those that occur at the lower rates seen in space [1]. Animal experiments using multiple small dose fractions and/or very low-dose rates of densely ionizing radiation are needed to reduce uncertainties in predicting risk at space-relevant dose rates [34,35]. Likewise, evaluation of countermeasure efficacy may also depend on dose rate.

Thus, a significant challenge for space radiobiology research is developing strategies to simulate a chronic GCR exposure of up to 3 years, approximating a Mars mission, in relation to simulations with animal models of human risks. To more closely simulate the low-dose rates found in space, single-ion and mixed-field exposures can be divided into a large number of fractions over long periods of time (e.g., daily fractions over 2–6 weeks). Individual beam fractions as low as 0.1 to 0.2 mGy can be reliably measured and delivered at the NSRL (see NSRL facility modifications). The goal is to design a highly fractionated simulation scheme, within animal and facility operational constraints, such that the time scale of a biological response becomes primarily a function of dose (i.e., insensitive to dose rate) to more closely mimic the space environment. Computational analyses of accelerator GCR simulations have shown that a large percentage of cells will be hit with 2 or more particles in simulated chronic exposures of a week or less and recommend exposures of several weeks with times approaching 30 days or longer to avoid any high dose-rate artifacts [36,37]. Likewise, considering the relative life span of humans compared with mice, in which 32 human days are estimated to roughly scale to 1 mouse day [38], a duration of approximately 30 mouse days is suggested to mimic a 3-year human exploration mission duration. Thus, the first chronic GCR simulator studies will attempt to scale to both dose rate and life span.

NASA baseline chronic GCR simulations will expose animal models to highly fractionated doses for 6 days/week over a 2- to 6-week period. The seventh day of the week is reserved for contingency in case of unforeseen operational difficulties. Total exposures are 250 mGy (over 2 weeks; delivered in 12 fractions), 500 mGy (over 4 weeks; delivered in 24 fractions), and 750 mGy (over 6 weeks; delivered in 36 fractions). The total daily dose of 20.8 mGy and dose fraction from each ion group is fixed, allowing for 3 dose points delivered on the same fractionation schedule. As an example, for a 4-week chronic 500 mGy exposure each of the 33 beams (shown in Table 3) will be delivered daily in 24 equal fractions with proton doses delivered daily in 0.3 to 5.1 mGy fractions, He doses delivered in 0.1 to 1.0 mGy fractions, and HZE particle doses delivered in 0.17 to 0.49 mGy fractions. Future studies are planned that will utilize slightly different exposure schemes, e.g., the delivery of 250 mGy over 4 weeks. These fractionated schemes rely on the ability to repeatedly deliver small doses and impose additional constraints limiting the number of HZE particles selected to represent the design reference field. To improve our understanding of dose and dose-rate effects (DDREF), NASA guidance requires that chronic studies include an acute exposure at an equivalent dose. Given the continuous low-dose rates found in space (<1 mGy/day), challenges remain in simulating the galactic cosmic ray environment in Earth-based analogs [4]. Modeling is expected to play a key role in interpreting and translating biological results from acute (high dose rate) and fractionated exposures to the protracted [35] and mixed-field [30] exposures humans see in space.

Other parameters exist to approach a low dose-rate limit or a region of operations such that particle hits are independent on relevant biological timescales. Under these conditions, results should be scalable to the very low dose and dose rates of the space environment. Witihin the current implementation strategy, 3 parameters are adjustable: the dose fraction (mGy) of ions delivered, the dose rate of delivery (Gy/min), and the time between daily particle doses (from minutes to hours to days). Facility operations constrain the flexibility of solutions given: low-dose fractions must be reliably and repeatedly delivered and measured by NSRL dosimetry, the dose rate is limited by the spills per cycle as dictated by the operation of the Department of Energy’s Relativistic Heavy Ion Collider (RHIC) at BNL, and the time between the 33 daily particle doses is limited by the duration animals can be housed in the target room (typically 8–10 hours). Other schemes to approach a low dose-rate limit include the delivery of the GCR simulator on a different daily schedule, e.g., 3 times a week over a longer time course.

#### Simplified 5-ion GCR simulator

NASA has also defined a “Simplified 5-ion GCR Simulator” beam for use in the initial understanding of sequential-field quality effects, collection of preliminary data to power studies, as well as for use in countermeasure screening studies. The ion-energy beam combinations are defined in Table 6 and require less time to deliver compared with the full 33-ion GCR simulation. The order of beam delivery and dose fractions are consistent with the full GCR simulator (Table 3), with proton irradiation first and last in the sequence as well as delivering the majority of exposure. Although aspects of the spectral characteristics of the reference field are lost in not capturing the full LET dependence of the low-energy protons and helium components, the selected beams will provide uniform dose profiles within both mice and rats given that the ion ranges greatly exceed animal tissue thicknesses. This is also the case with larger rats given that they remain flank to the incoming beam (i.e., not nose to tail).

**Table 6:** Simplified 5-ion mixed field normalized to 500 mGy.

Ion species	Energy (MeV/n)	LET (keV/ μm)	Range (cm)	Dose (mGy)	Percent contribution to total dose (%)	delivery order	Fractionated dose- 24 exposures (mGy/day)
^1^H	1,000	0.2	326.6	174.1	35	1	7.3
^28^Si	600	50.4	22.7	5.7	1	2	0.2
^4^He	250	1.6	38.3	90.2	18	3	3.8
^16^O	350	20.9	16.9	29.1	6	4	1.2
^56^Fe	600	173.8	13.1	5.1	1	5	0.2
^1^H	250	0.4	38.1	195.9	39	6	8.2
			total	500.0			20.8

**Abbreviations:** LET, linear energy transfer

While the full NSRL GCR Simulator is being used to deepen our understanding of well-characterized model systems and to test hypotheses related to mixed field and chronic (highly fractionated) exposures, studies of relatively less mature model systems that lack deep historical data sets of single ions are generating research results using the simplified field. Results from the simplified field support the development of predictive models in which our understanding of ion dependency/interdependency is limited, especially with respect to potential CNS decrements. The shorter time for acute and chronic irradiations can increase NSRL throughput of animals, especially in the case of rat studies, in which each cave entry to the target room is limited to 15 animals for the large beam configuration. Likewise, constraints on cell culture systems may also benefit from the shorter irradiation times. Updates to the Simplified 5-ion GCR simulator will be based on experimental consortium results, mixed-field studies, and model predictions as well as results from the full 33-beam GCR simulator.

## Results

Operating the GCR simulator requires the ability to rapidly and reliably change between multiple ion-energy beam combinations and repeatedly deliver them to an experimental target at predetermined doses. Facility modifications, including both hardware and software efforts, supporting full GCR operations at the NSRL, were completed in early 2017. The system was commissioned at BNL in late 2017 and first used in 2018 for major PI-led research studies.

### Ethics statement

The Brookhaven Laboratory Animal Facility has been accredited by the Association for the Assessment and Accreditation of Laboratory Animal Care (AAALAC) since 1966. It follows the guidelines and regulations set forth in the “Guide for the Care and Use of Laboratory Animals,” which is enforced by the Office of Laboratory Animal Welfare (OAW Animal Welfare Assurance Number D16-00067 [previously: A3106-01]). In addition, all research involving animals must comply with the Public Health Service “Policy on Humane Care and Use of Laboratory Animals.” Furthermore, each research team must describe the specific details of their proposed studies in a BNL Institutional Animal Care and Use Committee (IACUC) protocol and receive official approval from the BNL IACUC before beginning any such activities. The animal work described in this report was approved by BNL (protocol numbers 417, 489, and 490), Colorado State University (OAW Animal Welfare Assurance Number A3572-01, protocol number 18-7851A), the University of Texas Southwest Medical Center (OAW Animal Welfare Assurance Number D16-00296 [previously: A3472-01], protocol number 2015–101305), and the University of Rochester School of Medicine and Dentistry (OAW Animal Welfare Assurance Number D16-00188 [previouslyA329201], protocol number 102221).

### NSRL facility modifications

**Fig 11:**
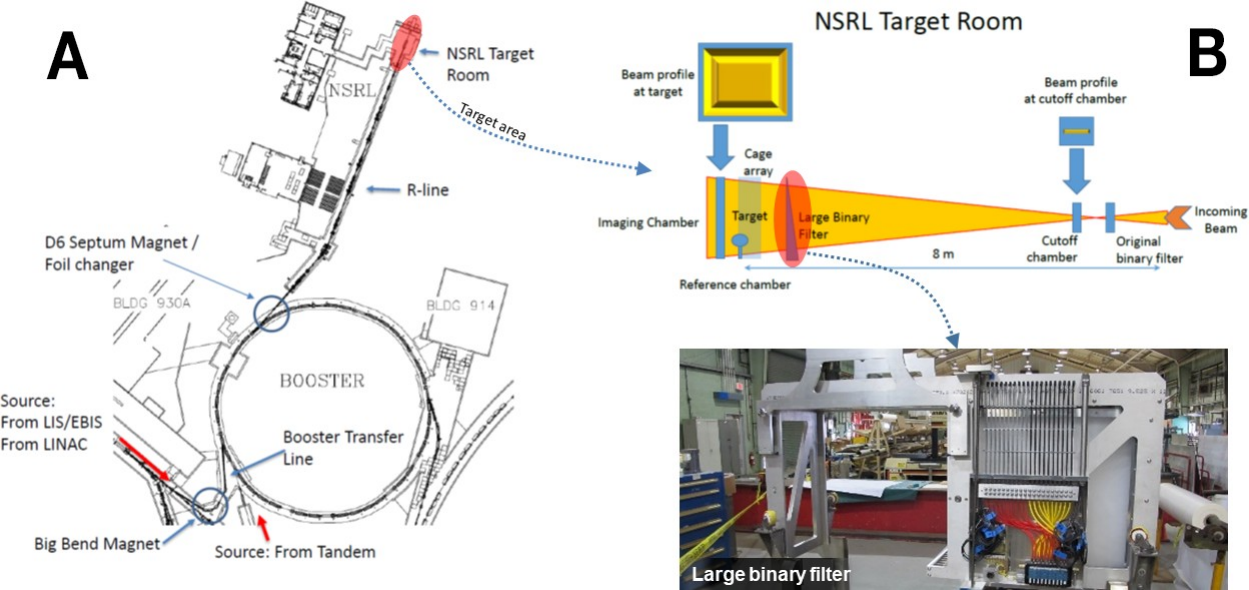
Facility layout of NSRL at BNL. (A) Tools to reliably control system hardware settings, from ion production by the LIS through booster injection, acceleration, extraction, and delivery to the NSRL target room were developed to sequentially deliver the GCR simulator ion-energy beam combinations. (B) Position of imaging chamber behind target (top, left-hand side), cut-off chamber (top, right-hand side) near beam entrance to target room, and photo of large-area degrader (binary filter) system (bottom) in NSRL beam line to maintain control and uniformity of 60 × 60 cm^2^ beam. BNL, Brookhaven National Laboratory; EBIS, Electron Beam Ion Source; GCR, galactic cosmic radiation; LINAC, Linear Accelerator; LIS, laser ion source; NSRL, NASA Space Radiation Laboratory.

Investments in advanced ion-source technology and control systems were essential to enable a mixed-field irradiation capability. GCR simulator ion beams are extracted from BNL’s Booster synchrotron and transported to the shielded target room within the NSRL facility as shown in Fig 11. Three sources can supply ions to the Booster, including the Linear Accelerator (LINAC; protons only), the Tandem Van de Graaff (ions and protons), and the EBIS (any ion except protons) [39]. The GCR simulator relies on EBIS for helium and heavy ions and either the LINAC or Tandem for protons. EBIS has 2 ports for gas sources that are manually changed to switch between ion species generated. Helium occupies one of those ports during GCR simulations. In late 2013, EBIS was equipped with a laser ion source (LIS), which has the capability to produce ions from many different solid targets as well as to rapidly switch between solid-source targets through automated controls software. Modifications to fit the LIS’s vacuum chamber with a translation table enabled the installation of the specific solid targets needed for the production of ^12^C, ^16^O, ^28^Si, ^48^Ti, and ^56^Fe required by the simulator. With these control modifications and helium gas provided as a source, ion and energy changes are achieved by a single command in less than 2 minutes [39]. In late 2016, a variable thickness stripping foil changer was installed at the upstream end of the D6 septum magnet to improve control of source-beam optics after Booster extraction and prior to injection into the NSRL beam line. The foil changer consists of 8 individual thicknesses of foils from 0.25 to 16 mil (6.35 × 10^−4^ cm to 4.064 × 10^−2^ cm) with the thinnest foils made of aluminum and the thicker foils (≥1 mil or ≥2.54 × 10^−3^ cm) made of copper which are remotely controlled in the beam line to provide a range of thicknesses from 0.25 to 63.75 mils (6.35 × 10^−4^ to 1.619 × 10^−1^ cm). This upgrade from the previous 2 foil filter system enables better control of the beam shape at the target location in switching from one ion-energy combination to another [39]. In the summer of 2021, EBIS II will be installed replacing the current EBIS and provide the capability for direct gas injection into the beam line. Initial feasibility tests have provided confidence that EBIS II will have the capability to deliver protons to the NSRL and reduce the dependence on LINAC or Tandem as sources. Given the simulator delivers approximately 70% of dose from protons, this upgrade will provide an added level of autonomy and efficiency during operations.

The software controlling the order and dose of ion-energy beam combinations to the target is run locally from the NSRL dosimetry console. After ion production at the source, all hardware control settings along the beam lines, including moving species through the EBIS, the transfer line to the Booster (including the big-bend magnet), the Booster itself (injection and extraction), and the NSRL beam line (R-line), must be able to change to sequentially deliver different beams within a reasonable amount of time (Fig 11). A single software application was developed and extensively tested to reliably move from one hardware setting to another. The simulator control file contains the sequential list of ion-energy-dose combinations (Table 3) to which various elements of the accelerator and dosimetry systems must respond in a precisely choreographed sequence. A significant challenge was developing the software architecture to archive and recall the unique hardware control settings for each beam. The ion-energy beam combinations are selected and controlled by the BNL complex-wide Linux cluster while a NSRL local software program controls dose delivered using essentially a dose-based beam cut-off system. Because there are many opportunities for any part of the system to fail at any given time, a means to exit the sequence without overexposing the targets is essential. This was challenging and added much complexity to the software control system. Incremental demonstration of the modified system was successfully demonstrated in 2016 with the delivery of H, Si, and Fe ions to the NSRL target room in a single day.

As described previously (see Dose-rate studies), chronic GCR simulations require the delivery of individual beam fractions as low as 0.1 to 0.2 mGy. This represented another significant challenge for operations. In 2017, a series of tests were performed to demonstrate NSRL’s capability to image the beam at such low-dose rates, to retain sufficient monitoring and control over the beam shape, and to reliably measure the low daily doses specified for each beam. The Booster is a synchrotron, so the beam is delivered in “spills,” which last a few seconds. However, the beam can be terminated at a specified dose at any time during a spill. Located behind the biological target (or behind the cage array) is a digital imaging chamber capable of detecting doses as low as 0.05 mGy per beam spill. A cut-off chamber is positioned to intercept the beam at the upstream end of the optical bench or experimental rail system (see Fig 11B), where the beam area is smaller and the dose rate is about 10 times higher. At this location, the cut-off chamber can detect and reliably measure the beam. The cut-off chamber is calibrated against an NIST–(National Institute of Standards and Technology) traceable thimble ion reference chamber placed at the downstream end of the optical bench (in front of the imaging chamber) at the target location. The thimble ion reference chamber has also been verified against scintillation counters with various ions and energies to confirm fluence-dose relationships of delivered beams. Thus, dose calibration is transferred from a sensitive reference chamber at the target location, where the dose rate is low, to a chamber that can cut the accelerator beam off, located where the beam is much more intense. These dosimetry systems were tested at the extremely low doses required for chronic simulations confirming reliability and control over beam parameters.

The GCR simulator can be delivered in a 20 × 20 cm^2^ or in a larger 60 × 60 cm^2^ beam configuration. Typical dose rates of approximately 10 Gy/min and approximately 0.5 Gy/min are available for the 20 × 20 cm^2^ and the 60 × 60 cm^2^ configurations, respectively. Beam uniformity is monitored with segmented thin ion chambers and fluorescent screen techniques during beam preparation and dose delivery. The ion chamber is capable of measuring differences in intensity of 2% to 3% over the 60 × 60 cm^2^ area. During the 2018 GCR simulator runs, an older variable thickness degrader (binary filter) system was used to deliver the 20 lower energy proton and helium beams to the target from incident beams of 100 MeV/n. The original system was designed to be used with a beam size much smaller than 60 × 60 cm^2^ and was placed 8-m upstream of the target area to intercept the incident beam where its cross-sectional area was still small (Fig 11B). Because of this distance, the degraded beams arrived at the target with a nonuniform spatial distribution, peaking in the center and reduced to approximately 75% intensity at the corners. This represented a nonuniformity in overall dose of about 4% over the entire 33-beam cycle. During the first GCR simulations in the fall of 2018, animals in exposure cages were limited to the central portion of the large beam, leaving the corner cage boxes vacant, and were rotated through locations within the beam spot during fractionated runs.

In 2019, a large-area degrader system was installed approximately 1-m upstream of the target location to enhance spatial uniformity across the 60 × 60 cm^2^ beam spot (See Fig 11B). This now allows for the full utilization of the beam area and alleviates the need to rotate animal cage positions within the beam spot. The binary filter thicknesses are the same thicknesses as the previous system ranging from 0.1, 0.2, …, 12.8 cm of polyethlylene (with a density of 0.93 g/cm^3^). The resolution of this system enables verification of incoming beam energies through Bragg curve/range measurements, which is especially important for the lowest energy proton and helium beams utilized in the simulation. Because of its closer proximity to the target, these energy-degraded beams do not lose their shape and maintain good uniformity in dose, intensity, and calibration quality.

Prior to experimental runs, set-up and calibration files are tested several times to ensure the control systems are correctly reading files and providing required hardware settings. On a daily basis, operators confirm all ion beams are availble from the EBIS and run various ion-energy beam combinations (H_20_, He_20_, Si_600_, C_1000_, Ti_1000_, Fe_600_) to ensure the cut-off record is automatically created and that calibration and ion chambers readings are correctly recorded. These test runs help capture and track daily drifts in calibration, which are mainly caused by small drifts in beam shape and are typically within 4%. Other uncertainties, or errors, come from the beam cut-off system. The magnitude of the uncertainty depends on the dose delivered from a given ion-energy combination with larger percent errors associated with the smaller doses. Thus, this error is 0.1% to 2% for individual beams and about 0.2% for the cumulative dose delivered by the 33-beam cycle. These measures ensure the stability of the beam over long irradiation periods and over multiple days. Simulator set-up typically requires approximately 1 to 1.5 hours prior to daily experimental runs but can be much longer with unforeseen circumstances. Preparing for Simplified 5-Ion Simulator (Table 6) runs requires significantly less set-up time.

Each GCR simulation run requires 21 switches of unique ion-energy beam combinations to the target room. Switch times range between approximately 2 to 4 minutes with longer switch times required for He species to wait for the EBIS to clear prior to the introduction of a new species. The additional 12 beams (lower energy H and He) are generated via the binary filter in the NSRL beam line. Approximately 70 to 75 minutes is required to deliver the doses from all 33 beams for either acute (250 to 750 mGy) or daily fractionated exposures, which may pose a challenge for certain animal or cell model systems. The simplified 5-ion GCR simulation requires approximately 20 to 25 minutes to deliver either acute or fractionated exposures. Additional software records dose over each individual GCR simulation ion as well as cumulative dose for both acute and fractionated dose-rate protocols. Each investigator is provided with a detailed record of the absorbed dose in tissue and the duration of each radiation exposure.

### Animal and cell handling

**Fig 12:**
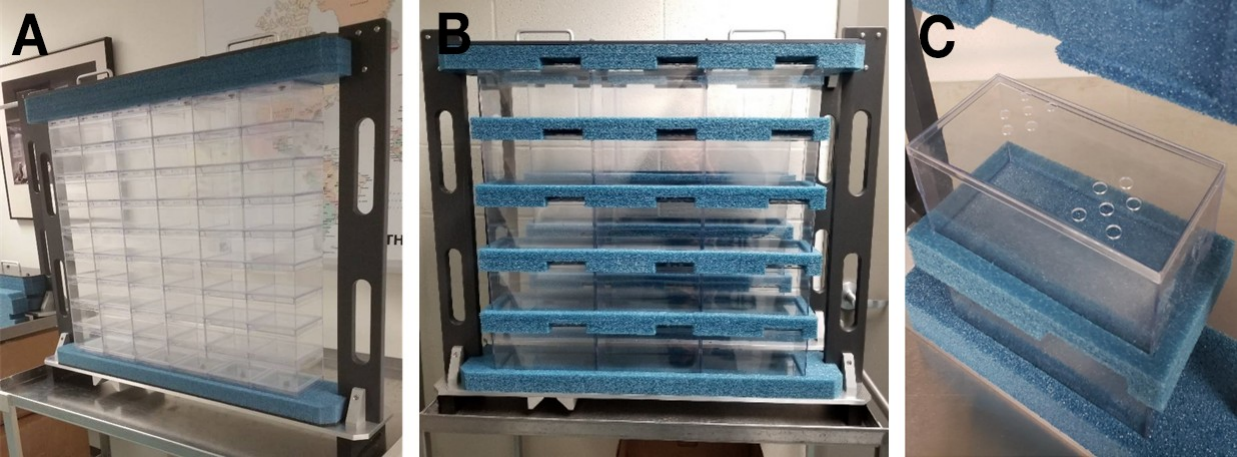
**Housing array for mouse (A) and rat (B) irradiations in the 60 × 60 cm**^**2**^ **beam.** Exposure boxes, made of approximately 2-mm thick polyethylene, stack together and are held in an array using a fabricated frame strucure. (C) Ventilation lids for air circulation are provided. The nonventilated sides of the lids are painted red to serve as a quick visual cue that the lids are in the correct orientation for air flow.

**Fig 13:**
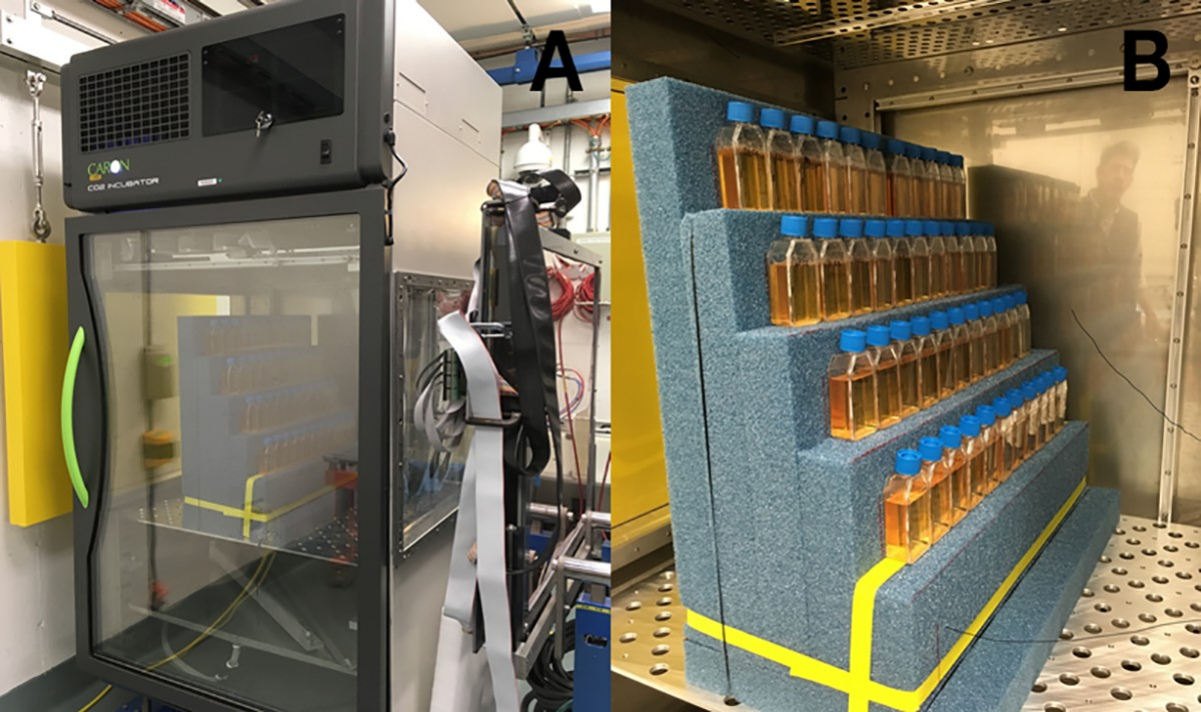
Modified incubator for use in beamline (A) with a holder that can accommodate up to 15 T75 flasks in a 3 × 5 array or 44 T25 flasks (B).

In the large beam spot (60 × 60 cm^2^), 54 special housing cages, configured in a 9 × 6 array, can accommodate at least 2 and sometimes 3 mice each (depending on age and genotype) for the 70 to 75 minute irradiation period (Fig 12A). The resulting range of subjects per exposure is 108 to 162 (with the high end of this range being very ambitious). The large beam can accommodate 15 special rat housing cages configured in a 3 × 5 array capable of holding 1 to 2 rats each depending on age, genotype, and orientation (Fig 12B). Special vents are provided in the tops of the cages with air circulation provided by external fans (Fig 12C). Blowing air across the loaded array while animals are waiting to be transported to the target room as well as during the irrradiation period is essential. An isopad is added in the bottom of the cage. For cell experiments, a large incubator has been modified for use in the 60 × 60 cm^2^ sized beam as shown in Fig 13A. Holders for both T75 and T25 flasks are available to experimenters (Fig 13B).

The cages for mouse exposures have interior dimensions of 10 × 10 × 4.5 cm in size providing plenty of space for movement. For acute irradiations, NASA guidance has limited the number of animals to 2 mice per cage or 1 rat per cage to avoid any ion range (Bragg peak) issues associated with body shielding from cagemates. For fractionated irradiations, the maximum cage capacity is limited to 3 mice and possibly 2 smaller rats. In this case, the positioning of the animals will be random during each daily fraction. Specialized rat cages have interior dimensions of 19.5 × 10.5 × 9.5 cm and also permit a significant amount of movement during exposures, even for the larger male Wistar rats weighing on the order of 700 to 800 g. The 19.5-cm length of the box is perpendicular to the beam to provide a lateral or flank exposure. Larger rats are unable to position themselves “nose to tail” in the beam direction. Based on a 90 minute dry run, with 2 Wistar rats, one rat turned 44 times in 90 minutes whereas the second rat turned 130 times in 90 minutes, thus exposing both flanks to the incoming beam direction. This active randomized movement helps provide homogenous dose distributions within larger animals during irradiations (see Analysis of dose distribution within animal models). The daily loading, exposing, and unloading of animals into and out of exposure boxes over an extended period may be stressful for some species. Thus prior to initiating the fractionated studies, dry runs of daily animal handling were performed over a 4-week period with a repesentative cohort of mice showing no signs of adverse effects.

### First GCR simulation research studies

The first radiobiology studies using the GCR simulator were performed by 3 PI-led research teams in 2018. Results from these studies will evaluate the effects of both acute and chronic doses on radiation-induced health risks using a variety of animal models including (1) Alzheimer disease pathology using male APP/PS-1 Alzheimer disease model mice (APPswe/PS-1dE9 transgenic mice) and their wild-type littermates (C57BL/6 background); (2) radiation-induced hepatocellular carcinoma (HCC) in C3H/HeNCrl mice; and (3) radiation-induced lung cancer in male and female wild-type and LA1 mutant (a lung cancer susceptible mouse) mice on a 129 background. In addition, several cohorts of these mice will be behaviorally and cognitively tested to evaluate potential radiation-induced decrements to the CNS. Two potential mitigating agents were tested: fluvastatin against late CNS effects and the anti-inflammatory, anti-oxidative radioprotector CDDO (a synthetic triterpenoid 2-cyano-3,12-dioxooleana-1,9(11)-dien-28-oic acid) against tumorigenesis.

**Fig 14:**
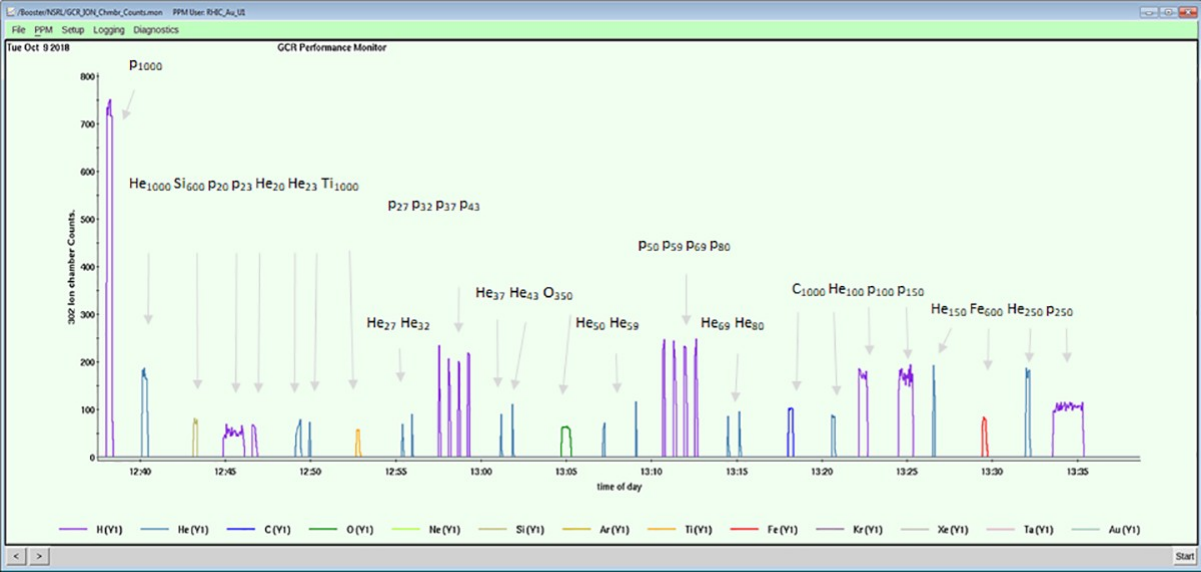
Computer screen shot measuring GCR simulator doses per particle for the 20.8 mGy cycle. GCR, galactic cosmic radiation.

Each study included an acutely exposed group, a fractionated exposed group with consecutive doses of 20.8 mGy/day delievered over a 4-week period, and sham controls. In efforts by NASA to standardize experimental methods, all 3 animal groups (acute, fractionated, and shams) were loaded, held in exposure boxes for 75 minutes, and unloaded daily from housing arrays to control for handling and restraint stress. While the simulator was basedlined to 500 mGy, the HCC study plan used a 400 mGy single exposure and a chronic 400 mGy exposure with 19 fractions delivered over 4 weeks (approximately 5 days a week) to compare directly with previous neutron and single-ion experimental results. The other teams irradiated cohorts with a single 500 mGy exposure and a chronic 500 mGy exposure with 24 consecutive doses of 20.8 mGy delivered 6 days a week over the 4-week period. Acute exposures were typically delivered halfway through the fractionated exposure to support age-matching post irradiation. During the fractionated simulations, cage boxes were moved daily through the 9 × 6 cage array, with exception of each of the 3 corner boxes, so that each animal sampled all parts of the beam by the end of the 4-week period. Likewise, for the acutely exposed groups, animals were not housed in the outer corner cage boxes of the array. With the increased beam uniformity achieved with the newly installed large binary filter (Fig 11B), this is no longer a requirement for future studies (see NSRL facility modifications). Recorded exposures times (either the acute or fractionated) were approximately 70 to 75 minutes with the largest portion of time devoted to beam switching. Fig 14 shows dose tracking of the individually delivered ions as viewed on the NSRL dosimetry console.

During this initial experimental campaign, over 500 mice were handled daily with approximately 300 mice receiving daily irradiations and approximately 200 mice treated as acute and sham controls. Based on camera footage from the target room, the mice adapted nicely to the exposure boxes and remained active especially with an isopad provided. This was the case even after being loaded into and out of the cages over the course of 4 weeks and alleviated concerns over the possibility of mice being shielded by cagemates while sleeping in the corners and not receiving a uniform dose in all directions. To avoid cross contamination from mixing cohorts, mice were handled in 3 distinct groups. For a given exposure, the beam preparation room and each loaded cage array only contained animals from a single PI team. Because the loading and unloading of cohorts require a significant amount of time, multiple housing arrays (Fig 12) were fabricated to enable efficient transitions from one team to the next. The NSRL 18 C (fall 2018) run was extremely important in establishing concept of operations for future studies and providing a realistic determination of set-up and run times for future schedule planning.

## Discussion

Past ion source and control technology at accelerator facilities limited decades of biological research to studying model systems acutely exposed to a single ion. These studies are expensive and require large cohorts of animals and/or time consuming cellular assays to obtain each ion-specific data set. Given the complexity of the space radiation environment, numerous studies spanning relevant ions and energies across multiple model systems and endpoints are required to quantify and mitigate radiogenic health risks faced by astronauts. Major scientific questions on the additivity of biological responses from single-ion data and the effect of dose rate remain.

- Is there synergy, antagonism, or neither in space radiation-induced tumorigenesis, cardiovascular disease, or CNS decrements after mixed GCR-like exposures compared to that predicted from results with individual ion beams?
- How should dose-rate modifiers be implemented in models of risk to scale from acutely exposed human terrestrial cohorts (low LET gamma) to astronauts who are chronically exposed to a mixed field of low- and high-LET ions?
- Will dose-rate scale risk inversely, conventionally, or have no effect?
- Can a ground-based analog operate in a low dose and dose-rate regime that sufficiently mimics the space environment to validate countermeasures?

Answers to these questions were previously inaccessible utilizing ground-based irradiation facilities. Space-based research platforms beyond LEO and outside Earth’s protective magnetosphere do not exist. Although flight studies are recognized as an important component in characterizing and mitigating radiogenic health risks, the scientific evidence is largely expected to be acquired on the ground. Until now, there were no ground-based facilities in the world capable of simulating the mixed-field GCR environment.

Major NASA deliverables supporting human exploration include models to quantify risk, development of PELs, and countermeasures to mitigate health risks. With decades of single-ion research spanning particle types and LET, NASA’s cancer risk model and cancer PELs are most mature [26,40]. However, important questions remain on the validity of simple additivity and use of dose-rate modifiers in estimating risk from chronic mixed-field exposures which better simulate the space environment. The development of risk models and validating PELs, in the units of Gy-Eq, for the risks of cardiovascular disease (CVD) and potential CNS decrements are less mature with radiation quality effects largely unknown [40]. GCR simulation research studies have the ability to accelerate early CVD and CNS PEL development using a mixed-field quality factor (QF) and dose-rate (DREF) modifier assessed in single studies rather than through a multitude of acute ion by ion exposures with the need to then assess and validate additivity and dose-rate modification. An equally important goal of the GCR simulator is to provide a space-relevant environment to accelerate biological countermeasure testing for risk mitigation. Countermeasure evaluation across multiple radiogenic risks, drug dosages, and ion types supposes a large test matrix of conditions which can quickly become cost prohibitive. NASA’s approach to targeting countermeasures with the ability to reduce multiple risks through reduction of common indicators, e.g., inflammation, and testing efficacy in the highly fractionated, mixed-field environment of the GCR simulator can significantly reduce costs and accelerate identification and validation efforts. Although it is recognized that single-ion studies are still important in quantifying risk dependence on LET (or Z and E) to gain mechanistic insight on the interaction of radiation at the cellular and tissue level and its dependence on radiation quality. This information may be especially important in generating hypotheses to inform countermeasure development. Although the GCR simulator does not represent the detailed LET spectra for all shield designs and time in solar cycle, differences in the internal spacecraft environment spectrum compared with the ability to simulate in a ground-based facility with discrete monoenergetic ion beams is deemed small in comparison [8].

Recognizing that implementation of the these studies require a significant investment of resources, NASA has developed a tissue share program across our PI pool to enhance secondary science objectives. Cross risk studies (Cancer, CVD, and CNS) utilizing the same irradiated cohorts have also been successfully formulated and implemented by NASA to increase return on investment. The availability of multiple ion beams to users on any given day has greatly benefited physics research, dosimetry, and electronics testing with enhanced schedule flexibility.

The GCR simulator reference environment and implementation strategy were baselined in 2017. As experimental results become available, updates to field parameters can be easily incorporated, including increasing the number of discrete ion and energy beams used or modifying existing ion-energy beam combinations. Given the success of the 2018 and 2019 simulator operations, potential updates can confidently include approximately 10 to 12 species of particles with Z ≥ 3 generated by the LIS. However, as the number of ions are increased, the dose delivered from each ion decreases and will be limited by the ability of the current NSRL dosimetry systems to reliably deliver and measure small doses, especially when moving to highly fractionated delivery schemes. With current operational capabilities, 0.1 to 0.2 mGy is the limiting low dose. However, other strategies to increase the number of representative HZE particles to generate a more continuous high-LET distribution may include moving to a different fractionation scheme where protons and helium are delivered daily while distinct HZE particles are delivered on a different schedule. For example, one group of HZE particles can be delivered on a Monday, Wednesday, Friday schedule while the second group of HZE particles are delivered on a Tuesday, Thursday, and Saturday schedule.

Other concepts have explored the use of absorbers in the beamline to attenuate and fragment heavy ions as a means to spread both the energy and fragment distribution to approximate the GCR environment found within spacecraft. Kim and colleauges, 2015 [36], suggest the use of simple slabs of polyethylene with 9 heavy-ion beams in addition to protons and helium, whereas Chancellor and colleagues, 2018 [41], suggest the use of a more sophisticated absorber in geometry and material selection with a single incident ion to simulate the complex mixture of nuclei and energies, including the secondary production of neutrons. As discussed, hybrid approaches are under consideration by NASA, which include the possibility of adding higher-energy incoming beams incident on specially designed absorbers/moderators in the beam line (Fig 5C).

Key simulation challenges remain in determining whether the delivered field of mixed ion species adequately approximates the GCR environment for radiobiology endpoints of interest, in understanding how best to implement dose-rate studies to better simulate chronic deep-space exposures, and in the selection of appropriate animal and cell models supporting translation to humans. Research results from early experiments are forthcoming and will inform future modifications to reference field specifications and implementation strategy for improved ground-based GCR simulations at the NSRL.

## Supporting information

S1 Data Data used to construct Fig 2, Fig 3, Fig 6, Fig 7, Fig 8 and Fig 10. (XLSX)

## Abbreviations

BFO: blood-forming organ
BNL: Brookhaven National Laboratory
CAD: computer aided design
CDDO: 2-cyano-3,12-dioxooleana-1,9(11)-dien-28-oic acid
CNS: central nervous system
CT: computed tomography
CVD: cardiovascular disease
DDREF: dose and dose-rate effectiveness factor
DREF: dose rate effectiveness factor
EBIS: Electron Beam Ion Source
EM: electromagnetic
GCR: galactic cosmic radiation
HCC: hepatocellular carcinoma
HZE: high charge and high energy ions
ICRP: International Commission on Radiological Protection
ISS: International Space Station
LEO: low Earth orbit
LET: linear energy transfer
LINAC: Linear Accelerator
LIS: laser ion source
MSL-RAD: Mars Science Laboratory radiation assessment detector
NIST: National Institute of Standards and Technology
NCRP: National Council on Radiation Protection
NSRL: NASA Space Radiation Laboratory
OLTARIS: On-Line Tool for the Assessment of Radiation in Space
PEL: permissible exposure limit
PI: principal investigator
π/EM: pions and electromagnetic radiation
QF: quality factor
RBE: relative biological effectiveness
REID: risk of exposure induced death
RHIC: Realistic Heavy Ion Collider
SPE: solar particle event
